# Microbial Odorant Detection Guides *Drosophila* Parasitoids Seeking Hosts in Fermenting Fruits

**DOI:** 10.1002/advs.75253

**Published:** 2026-04-13

**Authors:** Yueqi Lu, Lan Pang, Wenqi Shi, Ting Feng, Zhi Dong, Yifeng Sheng, Sicong Zhou, Longtao Yu, Hao Guo, Ying Wang, Jiani Chen, Jianhua Huang

**Affiliations:** ^1^ Zhejiang Key Laboratory of Biology and Ecological Regulation of Crop Pathogens and Insects Institute of Insect Sciences College of Agriculture and Biotechnology Zhejiang University Hangzhou China; ^2^ Ministry of Agriculture Key Laboratory of Molecular Biology of Crop Pathogens and Insects Zhejiang University Hangzhou China; ^3^ Key Laboratory of Agricultural Biosafety and Green Production of Upper Yangtze River College of Plant Protection Southwest University Chongqing China; ^4^ College of Life Science Institute of Life Science and Green Development Hebei University Baoding China

**Keywords:** host‐seeking behavior, microbial volatiles, odorant recseptors, parasitoid wasp

## Abstract

While parasitoid wasps have been extensively reported to rely on plant‐derived volatiles for host location, host habitats such as fermented fruits often harbor not only plants but also diverse microbial communities that are essential for hosts. This raises the question of whether parasitoid wasps exploit microbial volatiles for host location. Although some ecological evidence exists for such microbe‐mediated attraction, the underlying molecular mechanisms remain largely unknown. Here, using the *Leptopilina boulardi*–*Drosophila* *melanogaster* system, it is reported that yeast (a ubiquitous microorganism in host habitats) attracts both *Drosophila* hosts and *L. boulardi* female parasitoids. Four yeast‐derived ethyl ester volatiles that elicit olfactory attraction in female wasps are identified. Through integrated genomic and transcriptomic analyses, the complete odorant receptor repertoire of *L. boulardi* is characterized, and two female‐biased receptors (LbouOR167 and LbouOR136) are identified as necessary for the detection of these ethyl esters. Structural and functional analyses revealed that a conserved residue, Leu159, in both receptors is essential for odorant ligand binding. These findings uncover a microbial volatile‐mediated mechanism underlying parasitoid host‐seeking behavior and provide novel insights into the chemical ecology of a four‐party interaction involving fruit, fungi, *Drosophila* hosts, and their parasitoid wasps.

## Graphical abstract

Yeast microbes in fermenting fruits attract both host flies and their parasitoid wasps. Female *Leptopilina boulardi* detect yeast‐emitted ethyl esters via two olfactory receptors, LbouOR167 and LbouOR136. A conserved residue, Leu159, is critical for binding these compounds, enabling female wasps to locate host‐rich habitats. This study uncovers the molecular basis of a four‐party interaction (fruit–fungus–fly–parasitoid) mediated by microbial volatiles.

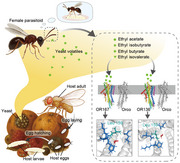

## 1 Introduction

Predator‒prey interactions play a pivotal role in shaping ecological communities. Successful predation often depends on a predator's ability to detect and locate prey via sensory cues [1, 2]. Among these methods, olfactory detection of prey‐derived chemical odorants is particularly important and has been widely exploited by diverse predatory species [3]. For instance, the wandering albatross, *Diomedea exulans*, relies on olfaction to detect fish‐derived odorants, enabling prey localization in pelagic environments [4]. Similarly, the arctic fox, *Vulpes lagopus*, uses its highly developed olfactory sense to locate frozen lemmings or subnivean seal lairs beneath packed snow [5, 6]. In addition, invertebrate predators also exploit chemical cues; for example, the predaceous bug *Elatophilus hebraicus* forages by detecting the sex pheromones of pine bast scales [7], while two zodariid spiders, *Zodarion rubidum* and *Habronestes bradleyi*, recognize ant‐derived chemical signals (trail and alarm pheromones) to locate prey [8, 9].

Parasitoid wasps (also known as parasitoids) are a specialized group of predators whose immature stages develop on or within their prey (typically other insect species, referred to as hosts), ultimately leading to host mortality [10]. Parasitoid wasps employ a hierarchical host‐searching strategy: they first locate the host habitat from a distance using habitat‐derived chemical cues, followed by short‐range host localization using host‐specific cues [11, 12, 13, 14, 15]. Substantial evidence indicates that long‐range host habitat localization generally involves the detection of volatile organic compounds (VOCs) emitted by either healthy plants or plants infested by herbivorous hosts [10, 16, 17]. For example, the parasitoid *Spathius agrili* locates the habitat of its emerald ash borer hosts by detecting the VOCs emitted by ash trees [18], while *Cotesia marginiventris* uses herbivore‐induced plant VOCs from maize to seek out cotton armyworm larvae hosts [19]. Similarly, *Cotesia popularis* finds its *Tyria jacobaeae* hosts via VOCs from damaged *Jacobaea vulgaris* [20], and *Trichogramma japonicum* is guided by a blend of volatiles from rice plants infested by its host, *Chilo suppressalis* [21]. In addition to plant volatiles, host habitats often harbor diverse microbial communities [22, 23]. Recent studies suggest that microbial volatiles (e.g., from bacteria or fungi) can mediate insect attraction in ecological settings [24, 25, 26, 27], raising the possibility that they may also play a role in guiding parasitoid wasps to their host habitats. Indeed, growing evidence supports this hypothesis across different parasitoid–host systems. For instance, the egg parasitoid wasp *Trissolcus basalis* is attracted to volatiles emitted by nectar fermented by specific bacterial strains, including *Staphylococcus epidermidis*, *Terrabacillus saccharophilus*, *Pantoea* sp., and *Curtobacterium* sp [28]. Similarly, females of the parasitoid wasp *Anagyrus dactylopii* exploit bacterial VOCs from *Kocuria rosea* and *Staphylococcus pasteuri* to locate their mealybug hosts, including *Maconellicoccus hirsutus* and *Nipaecoccus viridis* [29]. Furthermore, filamentous fungi isolated from the floral nectar of buckwheat (*Fagopyrum esculentum*) have been shown to alter nectar scent profiles and modulate the olfactory responses of two egg parasitoid wasps, *Trissolcus basalis* and *Ooencyrtus telenomicida* [30]. Despite these accumulating examples, the molecular mechanisms by which parasitoid wasps detect such microbial cues remain largely unexplored.

The *Drosophila*–parasitoid wasp system serves as a powerful model for dissecting the molecular mechanisms underlying ecological interactions and evolutionary adaptations [31, 32, 33]. Among these parasitoid wasps, those belonging to the genus *Leptopilina* (Hymenoptera: Figitidae) are particularly well‐studied due to the availability of rich genomic resources [33]. Within this genus, *Leptopilina boulardi* and *L. heterotoma* represent two extensively characterized species that preferentially parasitize *Drosophila* host larvae, with adult wasps emerging from the host puparium. Despite their similar morphology, body size, and generation time, these two species exhibit striking differences in host range: *L. boulardi* is a specialist that successfully parasitizes *D. melanogaster* and its close relatives, whereas *L. heterotoma* is a generalist capable of infecting a broad range of *Drosophila* species [34]. Importantly, *Drosophila* hosts themselves preferentially oviposit and develop in fermented fruits dominated by yeast communities, as yeast serves as a critical nutritional resource supplying essential nutrients including sterols, vitamins, and amino acids [35, 36, 37, 38]. Given the critical ecological dependence of *Drosophila* hosts on yeast communities in their natural habitats, it is noteworthy that the parasitoid wasp *L. heterotoma* has been shown to exploit yeast‐derived odors as a host location strategy to enhance its parasitic success [39]. In this study, we used the *L. boulardi*–*D. melanogaster* system to investigate two key questions: (1)  Do *Leptopilina* parasitoid wasps share a conserved strategy of utilizing yeast‐derived odors for host localization? (2) What molecular mechanisms govern this microbe‐mediated host localization behavior?.

## 2 Results

### 2.1 Yeast‐Derived Odor Attracts Both *Drosophila* Hosts and Their Parasitoids

**FIGURE 1:**
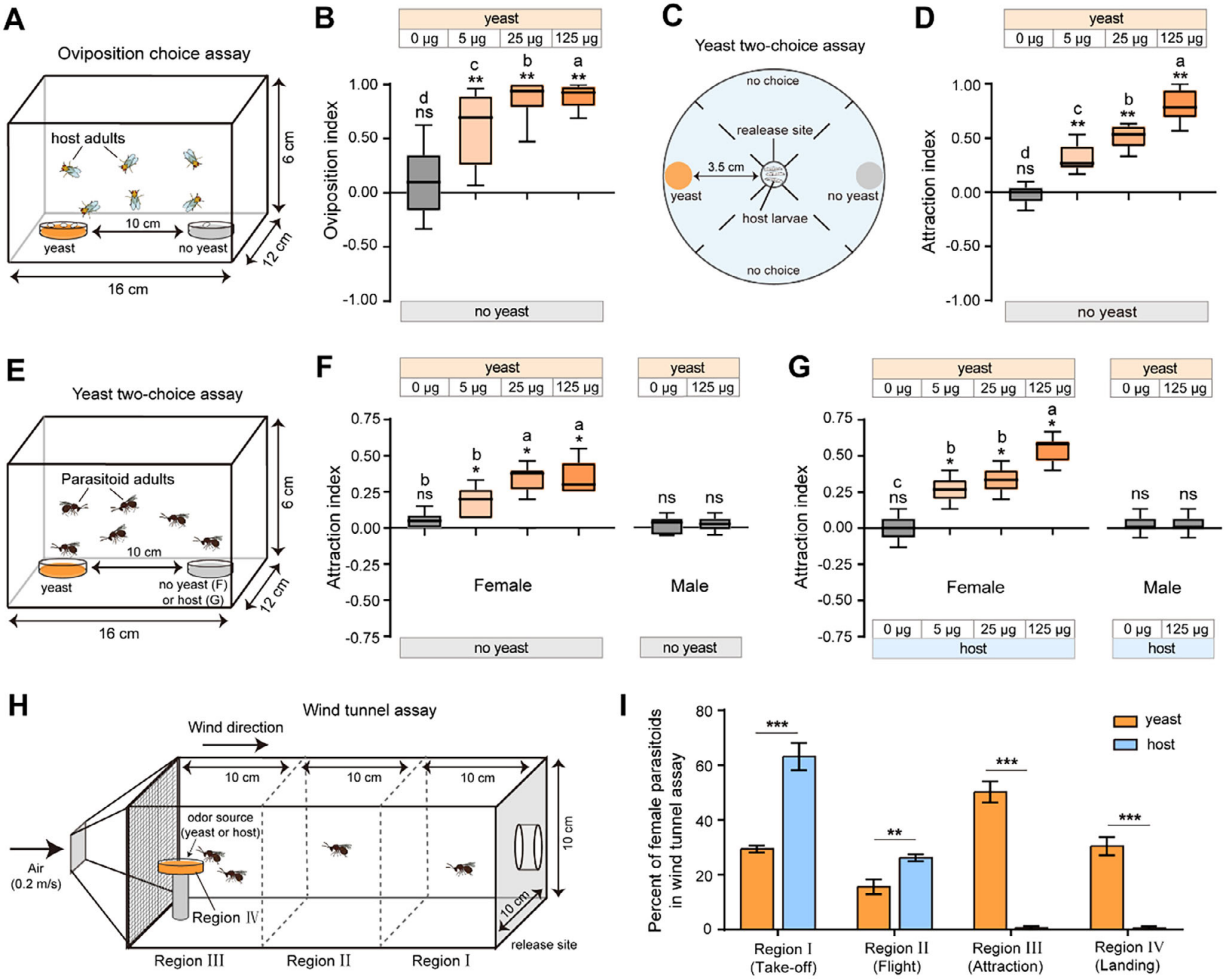
Both *Drosophila* hosts and *L. boulardi* parasitoids are attracted to yeast. (A) Schematic diagram of the oviposition assay for adult *D. melanogaster* females. (B) Oviposition preference of *D. melanogaster* hosts on yeast‐containing versus yeast‐free food plates. At least nine biological replicates were performed. (C) Schematic diagram of the two‐choice assay for *D. melanogaster* host larvae. (D) Attraction indices of host larvae to yeast in the two‐choice assay. Ten biological replicates were performed. (E) Schematic diagram of the two‐choice assay for *L. boulardi* parasitoids. (F) Attraction indices of female (left) and male (right) *L. boulardi* to different yeast quantities. At least six biological replicates were performed. (G) Preference of female (left) and male (right) *L. boulardi* for yeast versus host larvae. At least six biological replicates were performed. (H) Schematic diagram of the wind tunnel assay. Region definitions: I (take‐off), II (flight), III (attraction), IV (landing). Region III includes Region IV. (I) Distribution of female *L. boulardi* in response to yeast versus host larvae in the wind tunnel assay. Eight biological replicates were performed. In (B, D, F, and G) the box plots represent the median (centerline), interquartile (box), and full data range (whiskers), respectively. Significance against no preference (theoretical value = 0) was determined by one‐sample Wilcoxon's signed‐rank test (^*^ *p* < 0.05; ^**^ *p* < 0.01; ns, not significant). Differences among treatments were analyzed by one‐way ANOVA with Tukey's multiple comparisons test; different letters indicate statistically significant differences (*p* < 0.05). In (I) the data represent the means ± SEMs; significance was determined by an unpaired two‐tailed Student's *t*‐test or the Mann–Whitney *U*‐test (^**^ *p* < 0.01; ^***^ *p* < 0.001).

It has been shown that fermented fruits attract adult *Drosophila* females to lay eggs on them, an effect largely mediated by VOCs released by yeast [38, 40]. We designed an oviposition assay to examine the oviposition preference of adult *D. melanogaster* females between a yeast‐containing (yeast) food plate and a yeast‐free (no yeast) food plate (Figure 1A). As expected, a strong oviposition preference for the yeast plate was observed for *D. melanogaster* adults, with concentration‐dependent effects (Figure 1B). We next examined whether yeast‐derived VOCs also serve as attractants for *D. melanogaster* larvae using a two‐choice assay (Figure 1C). We found that *D. melanogaster* larvae exhibited significant attraction to yeast, with enhanced responses at higher yeast concentrations (Figure 1D). Consistent with previous findings, our results reveal that yeast‐derived VOCs function as effective attractants for both adult and larval *D. melanogaster*, consequently providing parasitoid wasps with abundant host resources.

Parasitoid wasps of the genus *Leptopilina* (Figitidae) are well known for parasitizing *Drosophila* host larvae [41, 42]. Notably, it has been shown that *L. heterotoma* exploits yeast‐derived odors as a host location strategy [39]. Here, we used the *L. boulardi*–*D. melanogaster* system to investigate whether *Leptopilina* parasitoid wasps share a conserved strategy of utilizing yeast‐derived odors for host localization. We employed a two‐choice assay similar to that used for the *D. melanogaster* oviposition preference tests, with a yeast plate versus no yeast plate (Figure 1E). Interestingly, we found that female *L. boulardi* were significantly attracted to yeast in a concentration‐dependent manner (Figure 1F). However, parallel experiments with male parasitoids revealed no such attraction to yeast‐derived volatiles (Figure 1F). These results suggest that yeast‐emitted odors might serve as host location cues for female parasitoids. To further test this hypothesis, we conducted another two‐choice assay in which a yeast‐containing (yeast) plate was compared with a host larva‐containing (host) plate (Figure 1E). Remarkably, female wasps consistently preferred yeast plates over those containing host larvae only, despite the latter providing short‐range host cues (Figure 1G). To verify such long‐distance attraction of *L. boulardi* to yeast volatiles, we next conducted wind tunnel assays to examine female wasp behavioral responses. Odor sources (yeast plate vs host plate) were positioned at the upwind end, with parasitoids released at the downwind terminus (Figure 1H). We quantified wasp distribution patterns across tunnel regions, categorizing their host location behavior from the initial take‐off phase to the final landing phase (Figure 1H, see Methods). When host larvae served as the odor source, parasitoid wasps were distributed primarily in regions I (take‐off phase) and II (flight phase), with almost no individuals demonstrating attraction (region III) or landing (region IV) to the plate (Figure 1I). In contrast, yeast volatiles elicited significantly stronger responses from female parasitoids, with substantially greater proportions of wasps reaching both the attraction zone (region III) and the landing zone (region IV) (Figure 1I). These results indicate that yeast‐derived VOCs serve as crucial cues enabling female parasitoid wasps to achieve successful host habitat localization.

### 2.2 Characterization of Yeast VOCs That Attract *L. boulardi* Parasitoid Wasps

**FIGURE 2:**
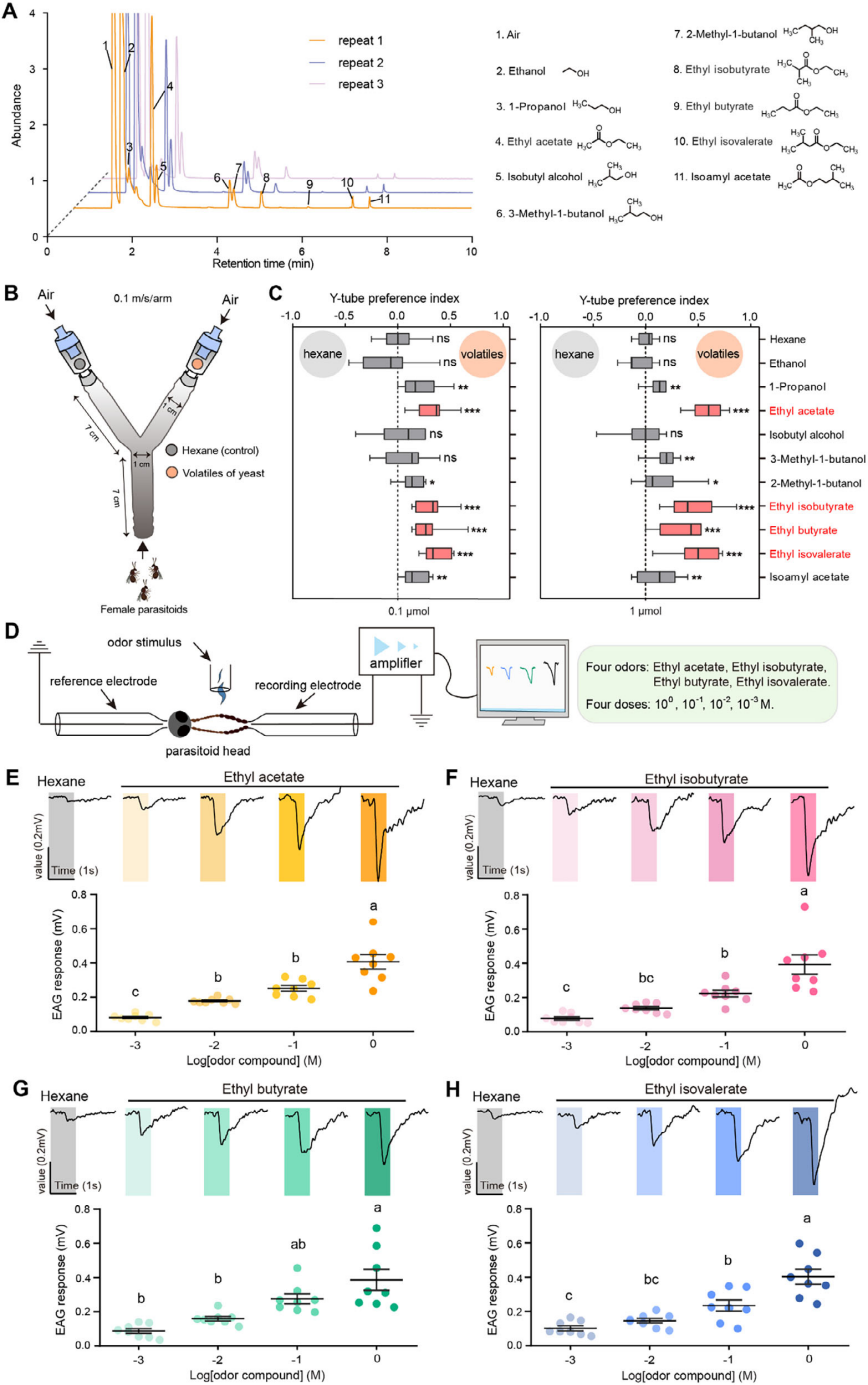
Yeast‐derived ethyl esters mediate the attraction of female *L. boulardi*. (A) GC‒MS profiles of yeast headspace volatiles. Peaks 1–11 correspond to the identified compounds (structures shown on the right). Three biological replicates were performed. (B) Schematic diagram of the Y‐tube olfactometer assay. Hexane served as the solvent control. (C) Preference indices of female *L. boulardi* in response to yeast volatile compounds at two dosages (0.1 and 1 µmol). At least eleven biological replicates were performed. Box plots represent the median (centerline), interquartile range (box), and full data range (whiskers). Significance relative to no preference (dashed line at 0) was analyzed by one‐sample Wilcoxon's signed‐rank test (^*^ *p* < 0.05; ^**^ *p* < 0.01; ^***^ *p* < 0.001; ns, not significant). Red boxes highlight compounds eliciting highly significant attraction (*p* < 0.001). (D) Schematic diagram of the electroantennogram (EAG) setup. Prepared *L. boulardi* female heads with antennae were subjected to odor stimuli delivered in a humidified airstream environment. Four ethyl ester compounds were tested at four doses. (E–H) Representative EAG traces and dose‒response curves of *L. boulardi* female antennae to ethyl ester compounds. The colored bars indicate stimulus pulses; the gray bars indicate hexane control pulses. Eight biological replicates were performed. The data are presented as the means ± SEMs. Significant differences were determined by one‐way ANOVA with Tukey's multiple comparisons test; different letters indicate significant differences (*p* < 0.05).

To identify the specific yeast volatiles that mediate *L. boulardi* female attraction, we performed a volatile analysis using gas chromatography‒mass spectrometry (GC‒MS). After the air peak was excluded, a total of 10 yeast‐derived VOCs were identified, which is generally consistent with the volatile profiles reported in earlier studies [39], including four ethyl esters (ethyl acetate, ethyl isobutyrate, ethyl butyrate, and ethyl isovalerate), 2 methyl‐butanol isomers (3‐methyl‐1‐butanol and 2‐methyl‐1‐butanol), ethanol, 1‐propanol, isobutyl alcohol and isoamyl acetate (Figure 2A). We next employed a Y‐tube olfactometer to screen the 10 identified compounds for their attractiveness to female *L. boulardi* wasps (Figure 2B). Each compound was tested at two different doses (0.1 and 1 µmol). Attraction of female parasitoids was observed in response to eight VOCs, with the strongest responses induced by four ethyl esters (ethyl acetate, ethyl isobutyrate, ethyl butyrate, and ethyl isovalerate) at both doses (Figure 2C).

We next conducted electroantennogram (EAG) recordings to monitor the antennal responses of parasitoids to the four ethyl ester stimuli (Figure 2D). We found that the parasitoid antennae exhibited strong, concentration‐dependent responses to all four compounds. Moreover, at the same concentrations, the antennal responses to these compounds were similar in magnitude. For instance, at the highest tested concentration, the EAG responses were 0.41 mV for ethyl acetate, 0.39 mV for ethyl isobutyrate, 0.39 mV for ethyl butyrate, and 0.40 mV for ethyl isovalerate (Figure 2E–H).

Collectively, the four yeast‐derived ethyl ester volatiles (ethyl acetate, ethyl isobutyrate, ethyl butyrate, and ethyl isovalerate) represent the key components in yeast volatiles that attract parasitoid wasps.

### 2.3 Comprehensive Identification and Phylogenetic Analysis of Odorant Receptors in *L. boulardi*

**FIGURE 3:**
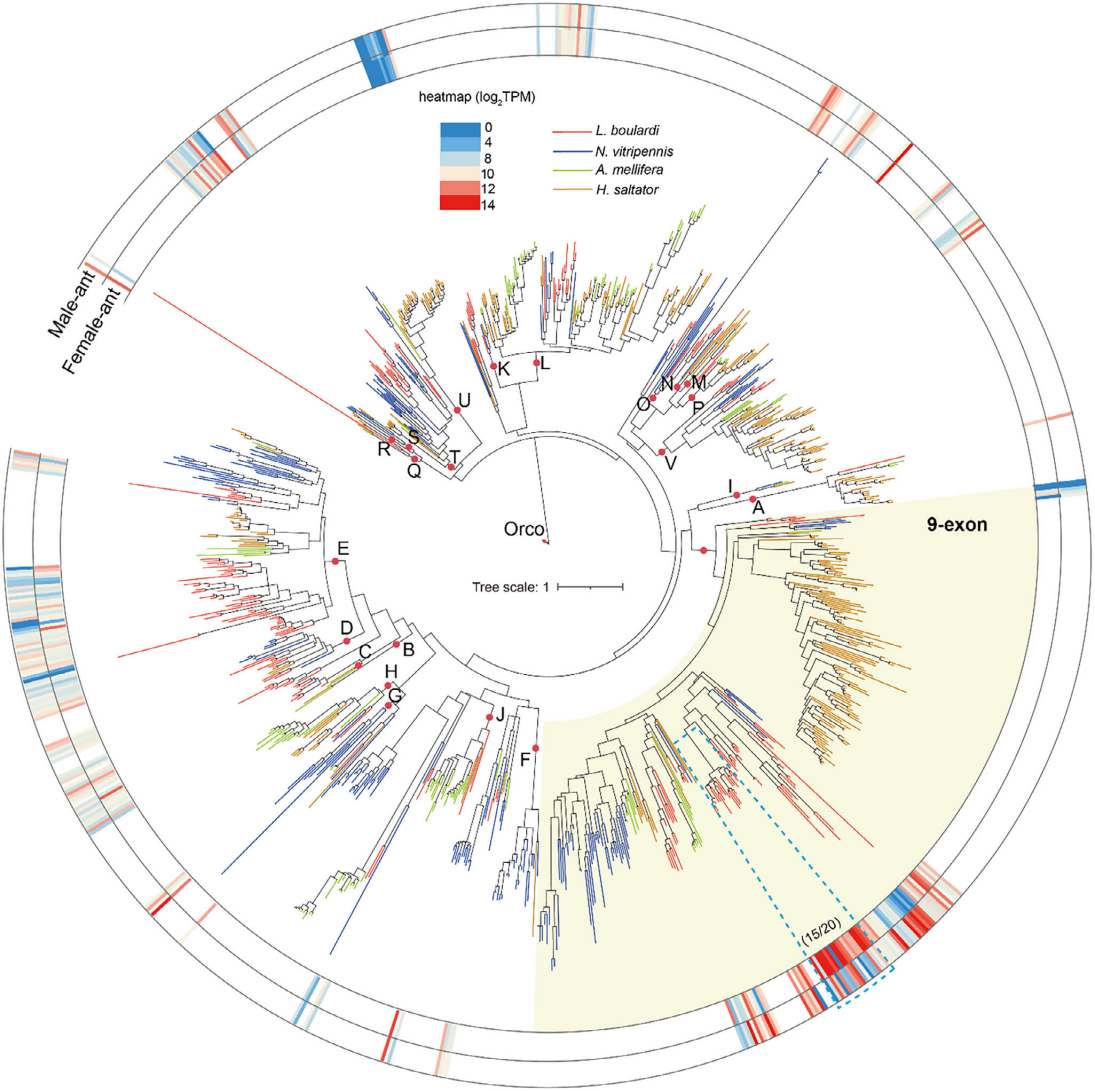
Phylogenetic analysis and sex‐specific expression profiles of *L. boulardi* olfactory receptors. Maximum likelihood phylogenetic tree of OR proteins from four hymenopteran species: *L. boulardi*, *A. mellifera*, *N. vitripennis*, and *H. saltator*. Red circles indicate well‐supported subfamilies. The 9‐exon subfamily is highlighted in yellow. Expression profiles of 238 *L. boulardi* OR genes in female and male antennae mapped onto the phylogenetic tree. The expression levels are represented by a blue‐to‐red color gradient (low to high). Gene clusters with female antennae‐biased expression in the 9‐exon subfamily are marked with a blue dashed frame.

*Odorant receptor* (OR) genes are expressed mainly in olfactory receptor neurons (ORNs) within olfactory appendages such as the antennae, where they are responsible for the perception of volatile chemical signals [43, 44]. Thus, we downloaded the publicly available *L. boulardi* genome and annotated the *OR* genes [45, 46]. In total, we identified 238 *L. boulardi OR* genes (*LbouORs*) in the genome (Table S1). Given the low sequence conservation of insect *OR* genes and their encoded protein sequences [47], we performed a comparative evolutionary analysis of ORs within hymenopterans using *L. boulardi*, *Apis mellifera* [48], *Nasonia vitripennis* [49], and *Harpegnathos saltator* [50] as representative species (Figure 3). In previous studies, hymenopteran ORs have been classified into 24 distinct subfamilies, including the A–V, Orco, and 9‐exon subfamilies [50, 51, 52]. Our phylogenetic analysis confirmed these findings but revealed variations in OR distribution among different hymenopteran species. Specifically, LbouORs were distributed across 13 subfamilies, whereas *A. mellifera*, *N. vitripennis*, and *H. saltator* possessed 18, 17, and 22 OR subfamilies, respectively. Among the four species, only the Orco subfamily exhibited strict single‐copy conservation. In contrast, five subfamilies (9‐exon, E, L, T, and V) underwent extensive gene expansion. Notably, the 9‐exon subfamily showed the most dramatic expansion in *L. boulardi*, *N. vitripennis*, and *H. saltator*, whereas the L subfamily experienced the greatest expansion in *A. mellifera* (Figure 3; Table S2).

Our behavioral assays demonstrated that *L. boulardi* females, but not males, exhibited a marked preference for yeast‐derived ethyl ester compounds. To elucidate the molecular mechanisms underlying this sexual dimorphism, we conducted comparative transcriptomic profiling of antennal *OR* gene expression between the sexes. Our analysis revealed 54 *LbouORs* with female‐biased expression and 43 with male‐biased expression (Table S3). Strikingly, the differentially expressed *LbouORs* were predominantly clustered within the 9‐exon subfamily, accounting for 28 female‐enriched and 15 male‐enriched receptors (Table S3). Notably, 15 of the female‐biased *LbouORs* were localized within a single rapidly evolving cluster of the 9‐exon subfamily (Figure 3; Figure S1). The exceptional concentration of female antennae‐specific *OR* genes in this cluster implies its critical role in mediating sex‐specific chemosensory behaviors, particularly those essential for host habitat localization.

### 2.4 Olfactory Receptors LbouOR167 and LbouOR136 are Required for Yeast‐Emitted Ethyl Ester Perception

**FIGURE 4:**
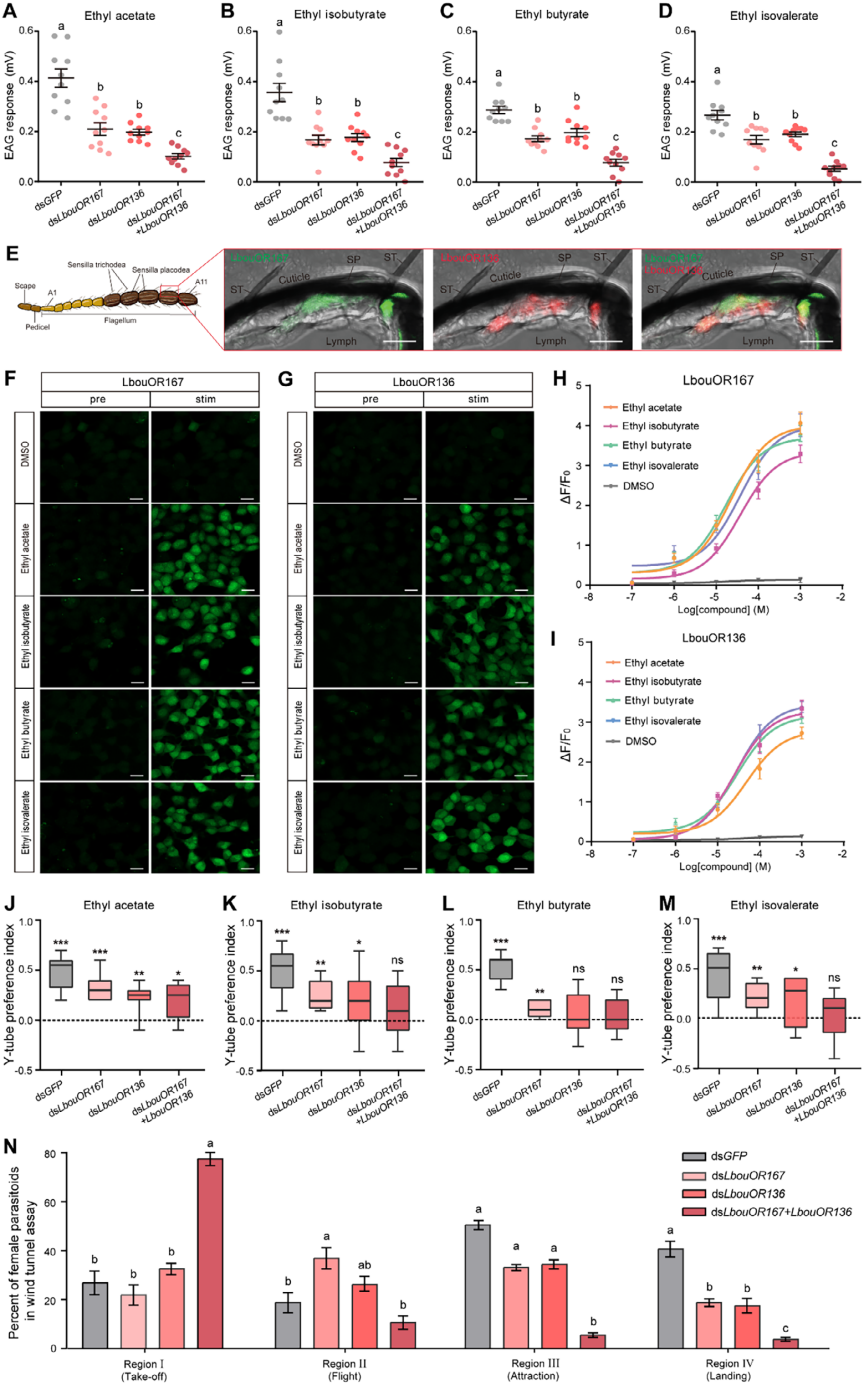
LbouOR167 and LbouOR136 are required for ethyl ester detection in *L. boulardi*. (A‐D) EAG responses of *L. boulardi* females to four ethyl ester compounds following RNA interference. Ten biological replicates were performed. Data represent the means ± SEMs. Significance was determined by one‐way ANOVA with Tukey's multiple comparisons test; different letters indicate significant differences (*p* < 0.05). (E) Localization of *LbouOR167* and *LbouOR136* in female *L. boulardi* antennae. The antennal structure consists of a scape, a pedicel, and a flagellum with 11 antennomeres (A1‐A11). Sensilla trichodea (ST) and sensilla placodea (SP) are distributed on the distal antennomeres. Scale bar: 10 µm. (F,G) Calcium imaging of HEK293 cells expressing *LbouOR167* or *LbouOR136*. Representative fluorescence images before (pre) and after (stim) stimulation with 10^−3^ m ethyl ester compounds. Scale bar: 20 µm. (H,I) Dose‒response curves of LbouOR167 and LbouOR136 to ethyl esters. Four to six biological replicates were performed. Data represent the means ± SEMs. (J–M) Preference indices of dsRNA‐treated *L. boulardi* females in Y‐tube assays between hexane and four ethyl ester compounds. At least eight biological replicates were performed. Box plots represent the median (centerline), interquartile (box), and full data range (whiskers). Significance relative to no preference (dashed line at 0) was analyzed by one‐sample Wilcoxon's signed‐rank test (^*^ *p* < 0.05; ^**^ *p* < 0.01; ^***^ *p* < 0.001; ns, not significant). (N) Behavioral responses of dsRNA‐injected *L. boulardi* females to yeast volatiles in wind tunnel experiments. Eight biological replicates were performed. Data represent the means ± SEMs. Significant differences were determined by one‐way ANOVA with Tukey's multiple comparisons test; different letters indicate significant differences (*p* < 0.05).

We employed the DREAM (deorphanization of receptors based on expression alterations in mRNA levels) approach [53]—a method that exploits the rapid changes in *OR* mRNA levels upon exposure to high ligand concentrations—to identify *ORs* responsive to four yeast‐derived ethyl ester volatiles, ethyl acetate, ethyl isobutyrate, ethyl butyrate, and ethyl isovalerate. Quantitative RT‒PCR (qRT‒PCR) analysis of the 10 most highly expressed female antennae‐specific *LbouORs* within the 9‐exon subfamily revealed significant downregulation of *LbouOR167* and *LbouOR136* mRNA levels after exposure to all four ethyl ester compounds (Figure S2). We next performed RNAi by injecting dsRNA targeting *LbouOR167* and/or *LbouOR136* into *L. boulardi* prepupae. qRT‒PCR confirmed the significant knockdown of both genes in emerging adult females (Figure S3). We observed significantly attenuated EAG responses to four ethyl esters in *dsLbouOR167*‐ and *dsLbouOR136*‐treated *L. boulardi* females compared with those in *dsGFP* controls, with the greatest reduction occurring in the double‐knockdown *L. boulardi* females (*dsLbouOR167*+*dsLbouOR136*) (Figure 4A–D). To investigate whether these two receptors function within the same sensilla, we performed in situ hybridization to determine their expression on female antennae and found that the transcripts of *LbouOR167* and *LbouOR136* were colocalized within identical sensilla (Figure 4E). Collectively, these findings suggest that the ability of LbouOR167 and LbouOR136 to detect ethyl esters might occur within the same olfactory sensilla.

To comprehensively validate the roles of LbouOR167 and LbouOR136 in perceiving yeast‐derived ethyl ester volatiles and regulating behavioral responses, we employed three additional experimental approaches: (1) heterologous expression, calcium imaging, (2) Y‐tube olfactometry, and (3) wind tunnel assays. First, we functionally expressed *LbouOR167* and *LbouOR136* in HEK293 cells with the Or‐coreceptor *LbouOrco* (Figure S4). Calcium imaging using a Fluo‐4 AM Ca^2+^ indicator revealed dose‐dependent responses to all four ethyl ester compounds in cells expressing either LbouOR167/Orco or LbouOR136/Orco but not in the solvent controls (Figure 4F–I). Second, Y‐tube assays with RNAi‐treated wasps revealed that compared with dsGFP controls, single knockdown of either *LbouOR167* or *LbouOR136* significantly reduced attraction to ethyl esters. As expected, double‐knockdown females exhibited nearly complete loss of chemotaxis (Figure 4J–M). Third, the results of the wind tunnel assays revealed a significant reduction in the proportion of parasitoid wasps reaching both the attraction zone and the landing zone for all the RNAi‐treated groups. Notably, the double‐knockdown group (*dsLbouOR167*+*dsLbouOR136*) exhibited the most severe behavioral deficits (Figure 4N). Collectively, these findings indicate that LbouOR167 and LbouOR136 are necessary for the molecular detection of yeast‐derived ethyl esters and host locations in *L. boulardi* wasps.

### 2.5 Molecular Modeling of Yeast‐Derived Ethyl Ester Volatiles Binding to LbouOR167 and LbouOR136

**FIGURE 5:**
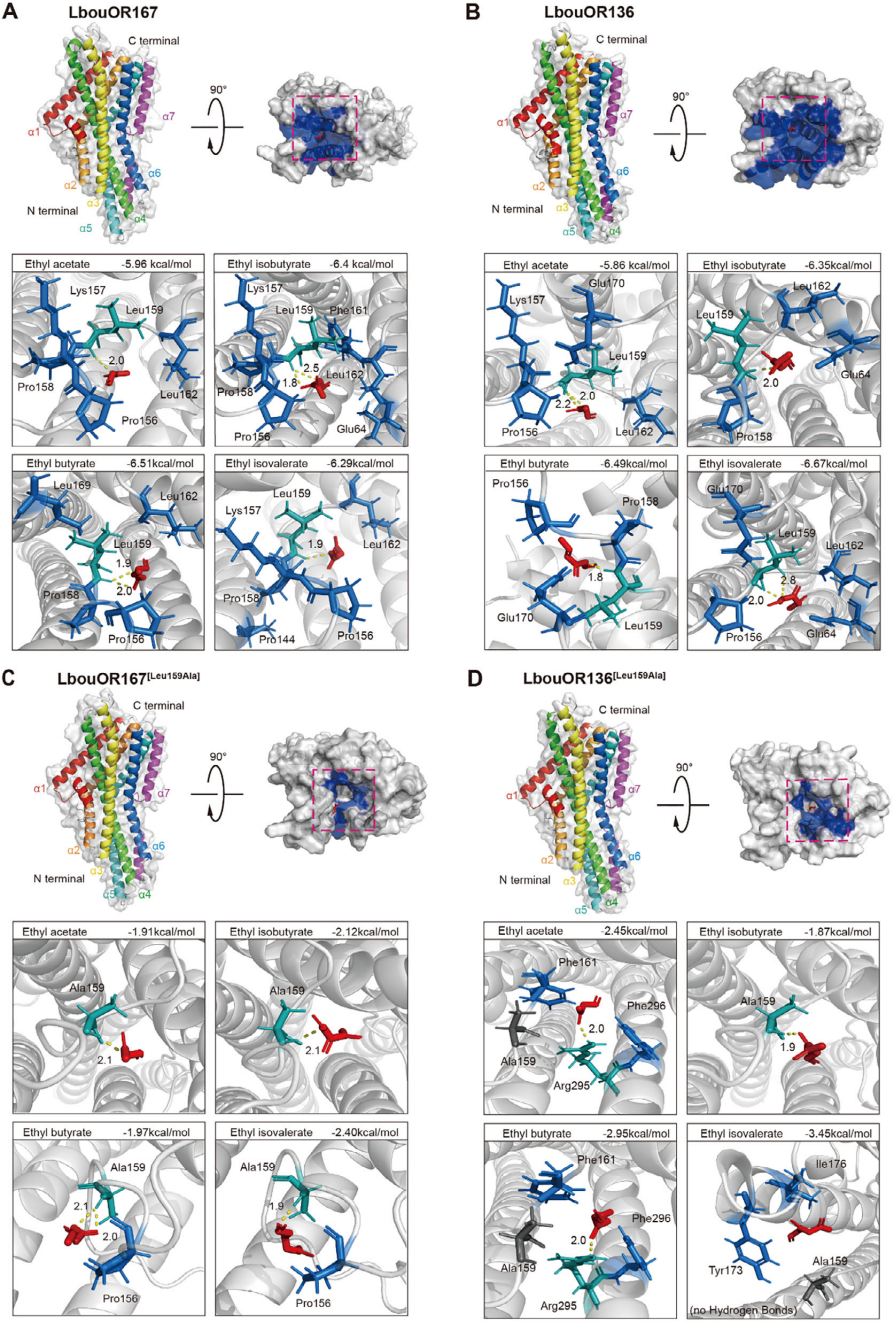
Structural models and molecular docking of LbouORs and their mutants with ethyl esters. (A) LbouOR167, (B) LbouOR136, (C) LbouOR167^[Leu159Ala]^, and (D) LbouOR136^[Leu159Ala]^. For each receptor, the predicted 3D structure (top) and molecular docking with four ethyl ester compounds (bottom) are shown. Top panels: Subunits are colored in a rainbow palette from the N‐terminus (red) to the C‐terminus (purple). Key α‐helices 1–7 are labeled. The gray surface represents the overall protein structure, and the blue surface represents the predicted binding pocket from an extracellular perspective. The approximate binding position of the ligand is outlined by a purple box, with ethyl acetate (red) shown as a representative compound. Bottom panels: The calculated binding energy values are provided. Ethyl ester compounds are shown in red. Key binding residues are colored in green, and hydrophobic residues are colored in blue. Yellow dashed lines indicate hydrogen bonds with corresponding distances (Å). In (D) dark gray indicates the Leu159Ala mutation site, which the backbone NH no longer participates in hydrogen bonding in the Ala variant.

To characterize the molecular interactions between four yeast‐derived ethyl esters (ethyl acetate, ethyl isobutyrate, ethyl butyrate, and ethyl isovalerate) and two parasitoid wasp ORs (LbouOR167 and LbouOR136), we employed protein modeling and molecular docking approaches. Using AlphaFold2 [54], we generated five 3D structural models for each receptor and selected the optimal models for LbouOR167 (Figure 5A) and LbouOR136 (Figure 5B) on the basis of maximal predicted local distance difference test (pLDDT) scores (Figure S5A,B). The optimal models exhibited canonical seven‐transmembrane α‐helical topologies with distinct extracellular loop architectures. Structural validation confirmed the high reliability of our models, with ERRAT analysis yielding quality factors of 93.67% for LbouOR167 (Figure S5C) and 96.85% for LbouOR136 (Figure S5D), whereas PROCHECK evaluation demonstrated that >97% of residues occupied energetically favored regions in the Ramachandran plots for both receptors (Figure S5E,F).

Molecular docking simulations of LbouOR167 and LbouOR136 with the four ethyl ester ligands revealed distinct binding pocket characteristics: LbouOR167 contained a 570.3 Å^2^ surface area (387.0 Å^3^ volume) with 48 residues, whereas LbouOR136 exhibited a larger 857.4 Å^2^ pocket (829.5 Å^3^ volume) comprising 63 residues (Figure 5A,B; Figure S6 and Table S4). Notably, both receptors showed strong binding affinities (ΔG ≤ −5.86 kcal mol^−1^) with all four ethyl esters, forming conserved hydrogen bonds with Leu159 (bond lengths ≤ 2.8 Å) along with significant hydrophobic interactions (Figure 5A,B; Table S5). These results suggest that Leu159 serves as a critical anchor residue for ethyl ester recognition, providing molecular‐level evidence that ensures that parasitoid wasps achieve host habitat localization.

### 2.6 Leu159 Residue is Important for Yeast‐Derived Ethyl Ester Recognition in LbouOR167 and LbouOR136

To investigate the role of the conserved Leu159 residue in yeast‐derived ethyl ester binding, we performed site‐directed mutagenesis (substitution of Leu159 with Ala) followed by structural and computational analyses. AlphaFold2‐generated models of LbouOR167^[Leu159Ala]^ and LbouOR136^[Leu159Ala]^ maintained structural integrity (Figure 5C,D; Figure S7) but exhibited markedly reduced binding pocket dimensions (LbouOR167^[Leu159Ala]^: 331.8 Å^2^ surface area, 101.4 Å^3^ volume; LbouOR136^[Leu159Ala]^: 393.6 Å^2^, 151.1 Å^3^) compared with those of wild‐type receptors (Figure 5C,D; Figure S8 and Table S4). Molecular docking analyses revealed that both LbouOR167^[Leu159Ala]^ and LbouOR136^[Leu159Ala]^ displayed reduced hydrophobic interactions with the ligands, with some ligand‒receptor combinations also showing diminished hydrogen bonding ability (Figure 5C,D; Table S5).

**FIGURE 6:**
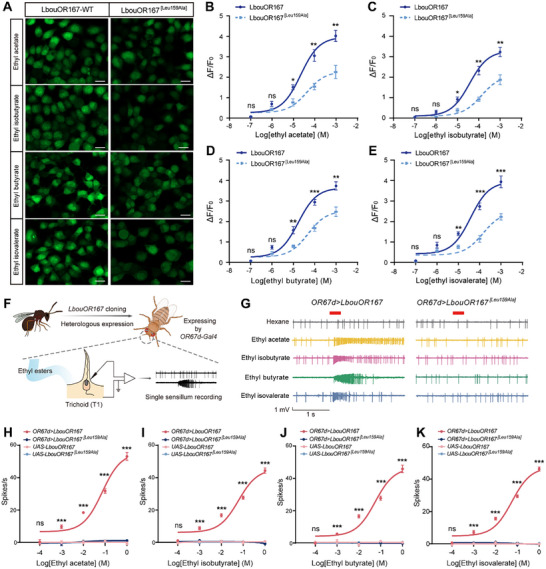
Mutation of Leu159 impairs ligand recognition and sensitivity in LbouOR167. (A) Representative calcium fluorescence images of HEK293 cells expressing *LbouOR167* or *LbouOR167^[Leu159Ala]^* after stimulation with 10^−3^ m ethyl ester compounds. Scale bar: 20 µm. (B–E) Dose‐response curves of LbouOR167 and LbouOR167^[Leu159Ala]^ to ethyl ester compounds. Four to six biological replicates were performed. Data represent the means ± SEMs. Significance was determined by an unpaired two‐tailed Student's *t* test (^***^ *p* < 0.001; ^**^ *p* < 0.01; ^*^ *p* < 0.05; ns, not significant). (F) Schematic diagram of the single sensillum recording (SSR) setup for the functional characterization of LbouOR167 and LbouOR167^[Leu159Ala]^ expressed in *D. melanogaster* Trichoid 1 (T1) neurons. (G) Representative electrophysiological traces from OR67d (Trichoid 1) neurons expressing *LbouOR167* and *LbouOR167^[Leu159Ala]^* when stimulated with 1 m ethyl ester compounds. (H–K) Response curves of flies expressing LbouOR167 and LbouOR167^[Leu159Ala]^ to ethyl ester compounds. *UAS‐LbouOR167* and *UAS‐LbouOR167^[Leu159Ala]^* flies were used as controls. *n* = 10 flies per concentration of each compound. Data represent the means ± SEMs. Significance between the responses of *OR67d > LbouOR167* and *OR67d > LbouOR167^[Leu159Ala]^* flies was determined by an unpaired two‐tailed Student's *t*‐test (^***^ *p* < 0.001; ns, not significant).

We then coexpressed *LbouOR167^[Leu159Ala]^* and *LbouOR136^[Leu159Ala]^* with *LbouOrco* in HEK293 cells and conducted calcium imaging using a Fluo‐4 AM Ca^2+^ indicator. Compared with cells expressing wild‐type receptors, cells expressing LbouOR167^[Leu159Ala]^ exhibited significantly attenuated responses to ethyl esters, with more pronounced impairment at higher ligand concentrations (Figure 6A–E). A similar reduction in calcium flux was observed for LbouOR136^[Leu159Ala]^ (Figure S9). For in vivo validation, we heterologously expressed *LbouOR167* and *LbouOR167^[Leu159Ala]^* into the Trichoid 1 (T1) ORNs of *D. melanogaster* using the *OR67d‐GAL4* knock‐in allele (Figure 6F). Single‐sensillum recordings revealed that T1 neurons expressing wild‐type LbouOR167 responded robustly to all four yeast‐derived ethyl esters in a dose‐dependent manner, whereas those expressing LbouOR167^[Leu159Ala]^ displayed significantly diminished responses that were nearly undetectable (Figure 6G–K).

Collectively, these findings establish Leu159 as a critical residue for ethyl ester recognition, confirming its predicted role in mediating ligand‒receptor interactions.

## 3 Discussion

**FIGURE 7:**
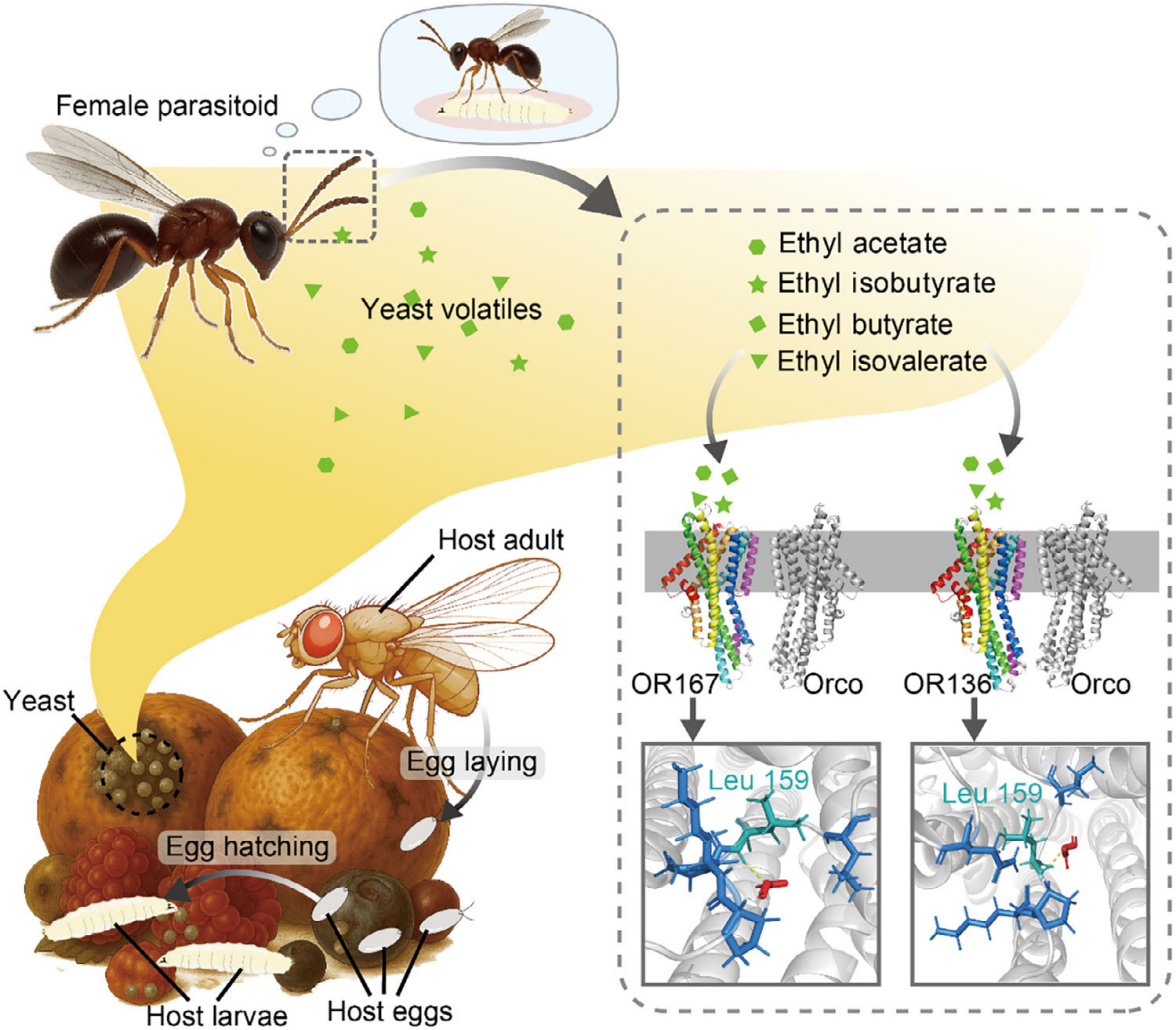
A model of microbial odor‐mediated host location in *Drosophila* parasitoids. Yeast, a key microbial component within the host habitat, attracts both adult and larval stages of *Drosophila* hosts. Female *L. boulardi* parasitoids detect yeast‐emitted ethyl esters (ethyl acetate, ethyl isobutyrate, ethyl butyrate, and ethyl isovalerate) via the olfactory receptors LbouOR167 and LbouOR136, which are essential for host habitat localization. The conserved residue Leu159 in both receptors is critical for binding ethyl ester compounds, enabling sensitive and selective olfactory responses that guide female parasitoids to host‐rich environments.

Parasitoid wasps are a highly diverse group of insects that are renowned for maintaining natural ecosystems through their unique parasitic strategies [55, 56]. Their parasitic success largely depends on an exquisite olfactory system that allows for parasitoid wasp females to detect chemical cues and locate suitable hosts [57, 58]. While previous research has emphasized plant‐derived volatiles in parasitoid host seeking [13, 16, 59, 60, 61], we and others reveal that parasitoid wasp females also exploit microbial volatiles to locate their hosts. This is particularly relevant in ecological contexts such as the fruit–fungus–*Drosophila* system, where yeast volatiles serve as reliable indicators of host‐rich habitats. Using the *L. boulardi*–*D. melanogaster* system, we further elucidated the molecular and structural basis of this microbe‐mediated host location process (Figure 7).

Olfactory recognition plays a critical role in predator‒prey interactions, particularly in parasitoid wasps. In insect olfaction, odorant molecules are initially captured by odorant‐binding proteins (OBPs) in the sensillar lymph, which shuttle them to olfactory receptors (ORs) on the dendritic membranes of olfactory sensory neurons, thereby triggering neural signaling cascades [62]. To identify ORs in *L. boulardi* that respond to yeast‐derived volatiles underlying host location, we annotated a complete repertoire of *OR* genes using a combined genomic and transcriptomic approach. We identified 238 *OR* genes in *L. boulardi*, a number consistent with those of other parasitoid wasps such as *N. vitripennis* (225) [49] and *Microplitis demolitor* (203) [63], and less than hymenopteran ants such as *Monomorium pharaonis* (306) [64], *Harpegnathos saltator *(347) [50], and *Solenopsis fugax* (314) [65]. In contrast, this repertoire is substantially larger than those of Diptera (*D. melanogaster*, 62) [66], Lepidoptera (*Manduca sexta*, 73) [67], and Hemiptera (*Rhodnius prolixus*, 129) [68]. This notable expansion of the OR family in Hymenoptera underscores its rapid evolutionary diversification, likely reflecting adaptations to specialized ecological niches and complex olfactory‐driven behaviors. The hymenopteran ORs have been categorized into 24 distinct subfamilies, among which the 9‐exon subfamily represents a hymenopteran‐specific lineage that has undergone remarkable expansion [50, 52, 69]. Our study revealed that the majority of female‐biased LbouORs belong to this subfamily. Functional investigations revealed two 9‐exon ORs in *L. boulardi*—LbouOR167 and LbouOR136—that respond to yeast‐derived ethyl ester volatiles (ethyl acetate, ethyl isobutyrate, ethyl butyrate, and ethyl isovalerate) and are involved in host localization by female wasps. However, how parasitoid females perceive host‐specific cues, such as cuticular hydrocarbons (CHCs), for short‐range host localization after arriving at the host habitat remains an open question worthy of further investigation. It is plausible that additional female‐biased 9‐exon LbouORs, beyond LbouOR167 and LbouOR136, contribute to these short‐range host recognition behaviors. Indeed, previous studies have demonstrated that 9‐exon subfamily ORs can detect CHCs that mediate social behaviors in hymenopteran insects, such as in the ant *H. saltator* [70]. In addition, the molecular basis underlying the sex‐specific response to yeast volatiles remains to be fully elucidated. Our transcriptomic data revealed that *LbouOR167* and *LbouOR136* are predominantly expressed in female antennae, with very low expression levels in males, consistent with the lack of behavioral responses observed in male wasps. Future studies are needed to determine whether this female‐biased expression pattern arises from haploid‐diploid sex determination or from other regulatory mechanisms.

Microorganisms are ubiquitous in nature, and emerging evidence indicates that microbial volatiles play a key role in mediating insect attraction within ecological systems. For instance, the nectar‐dwelling fungus *Metschnikowia reukaufii* produces scent compounds such as isoamyl alcohol, which attract moths such as *Mythimna separata* to pollen‐rich nectar, thereby enhancing their foraging efficiency and reproductive success [26]. The skin microbiota of flavivirus‐infected hosts emits acetophenone volatiles that facilitate mosquito attraction, consequently enhancing flavivirus transmission by mosquitoes [71]. In this study, we reported that the yeast *Saccharomyces cerevisiae*—a key microorganism in the *Drosophila* habitat [72]—simultaneously attracts both the parasitoid wasp *L. boulardi* and its host, *D. melanogaster*. Ethyl ester volatiles produced by this yeast, including ethyl acetate, ethyl butyrate, and ethyl isovalerate, have previously been reported to elicit olfactory responses in *Drosophila* hosts [73, 74, 75, 76, 77, 78, 79]. Here, we showed that female *L. boulardi* wasps were similarly attracted to these yeast volatiles, as previously reported for  *L. heterotoma* [39], whereas male wasps were not attracted to these volatiles. Given that yeast‐derived volatiles serve as potent attractants for both adult and larval *D. melanogaster*, they consequently provide parasitoid wasps with access to abundant host resources. As such, this female‐specific attraction suggests that *L. boulardi* has evolutionarily co‐opted the perception of yeast odorants as a host‐seeking strategy, enabling the efficient localization of host‐rich habitats and increasing parasitic success.

In *Drosophila* hosts, several ORs are known to detect yeast‐derived ethyl ester volatiles. For example, OR22a responds to ethyl acetate, ethyl butyrate, and ethyl isovalerate [73, 75, 78]; OR42a is sensitive to ethyl acetate and ethyl butyrate [76, 80]; and OR35a and OR42b are activated by ethyl butyrate [76]. In this study, we identified two ORs (LbouOR167 and LbouOR136) that respond to four yeast‐derived ethyl ester volatiles. Although these two receptors share 66% sequence similarity, they exhibit very low sequence homology (<20%) with their functional counterparts in *Drosophila* hosts. Furthermore, we determined that Leu159 serves as a critical residue for ligand binding in both LbouOR167 and LbouOR136. In contrast, key residues for yeast‐derived ethyl ester volatile binding in *Drosophila* OR22a include Ile45, Ile67, and Met93 [73]. Our results indicate that the recognition of conserved odorants does not necessarily require sequence similarity or homologous binding sites among odorant receptors. Our findings also provide a novel case of convergent evolution in olfactory mechanisms between a parasitoid wasp and its host, highlighting how distinct molecular strategies can underlie analogous ecological functions.

In conclusion, our study demonstrates that yeast‐derived ethyl ester volatiles serve as critical olfactory cues guiding host location in female *L. boulardi*. We identified LbouOR167 and LbouOR136 as the key receptors mediating this response, with structural characterization revealing that Leu159 is the essential binding residue for ethyl ester recognition. These findings provide mechanistic insights into how microbial volatiles influence parasitoid‒host interactions through specific chemosensory pathways while establishing a molecular framework for understanding predator‒prey chemical communication.

## 4 Experimental Section/Methods

### 4.1 Insects

*L. boulardi* parasitoid wasps were maintained on *D. melanogaster* hosts (Canton‐S strain) in our laboratory under the following conditions. Briefly, 50 mated *Drosophila* females were placed into a fly bottle containing fly food medium and allowed to lay eggs for 2 h. After the *Drosophila* eggs developed into second instar larvae, 10 mated parasitoid females were added to each bottle and allowed to parasitize the hosts for 6 h. The parasitized larvae were subsequently maintained at 25°C until the adult wasps emerged. Newly eclosed parasitoid wasps were collected and transferred to vials containing apple juice agar medium (27 g of agar, 33 g of brown sugar, and 330 mL of pure apple juice in 1000 mL of diluted water) for further experimental use. This medium is routinely used in our laboratory to maintain adult parasitoid wasps [42, 45, 46, 57]. Importantly, control experiments confirmed that wasps maintained on this diet exhibited behavioral responses to yeast volatiles comparable to those reared on sugar‐only diets (Figure S10), indicating that apple juice agar does not prime or enhance subsequent olfactory responses to yeast cues.

The *D. melanogaster* Canton‐S strain (#64349) was acquired from the Bloomington *Drosophila* Stock Center and was used in our previous study [42]. The *OR67d‐GAL4* line was originally obtained from Barry Dickson's laboratory at the Research Institute of Molecular Pathology, Vienna [58, 81]. To generate *UAS‐LbouOR167* transgenic flies, the coding sequence of *LbouOR167* was amplified from wasp cDNA using the primers listed in Table S6. The resulting cDNA fragment was digested with NotI (NEB, USA) and EcoRI (NEB, USA) and cloned and inserted into the pUAST‐attB plasmid. The Leu159Ala mutation was introduced into the *LbouOR167* pUAST‐attB plasmid using the Mut Express II Fast Mutagenesis Kit V2 (Vazyme, China) according to the manufacturer's protocol, with the primers listed in Table S6. Plasmids of *LbouOR167* and *LbouOR167^[Leu159Ala]^* were injected into *Drosophila* embryos and integrated into the attP site on chromosome 2 (cytology locus 25C6). All *D. melanogaster* strains were maintained on a standard cornmeal/yeast/sugar diet (recipe available at the Bloomington *Drosophila* Stock Center, https://bdsc.indiana.edu/information/recipes/bloomfood.html) at 25°C and 50% humidity.

### 4.2 Yeast

The yeast *Saccharomyces cerevisiae*, a ubiquitous microbe in host habitats, used in this study, was purchased from Sigma–Aldrich (#YSC2).

### 4.3 Oviposition Choice Assay

To determine whether adult *D. melanogaster* females preferentially oviposit on yeast‐containing food, we conducted two‐choice oviposition assays in transparent acrylic cuboid boxes (length: 16 cm; width: 12 cm; height: 6 cm; Figure 1A). Briefly, a fly food plate containing yeast (yeast) was placed on one side of the box, and a yeast‐free fly food plate (no yeast) was placed on the opposite side. Afterward, thirty mated 3‐ to 5‐day‐old female hosts were released into the apparatus and allowed to oviposit for 6 h. The number of eggs laid on each plate were subsequently counted. The oviposition preference index was calculated as (N_yeast_ − N_no yeast_)/(N_yeast_ + N_no yeast_), where N_yeast_ represents the number of eggs on the yeast‐containing plate and N_no yeast_ represents the number of eggs on the yeast‐free plate.

### 4.4 Yeast Two‐Choice Assay

To assess whether *D. melanogaster* larvae are attracted to yeast‐derived volatiles, we performed two‐choice assays in Petri dishes (diameter: 9 cm) filled with 1% agarose (Figure 1C). In brief, thirty *D. melanogaster* larvae were blotted dry on filter paper and then placed in the center of the dish. A filter paper disc (diameter: 0.6 cm) loaded with yeast was positioned on one side of the dish, and an identical disc without yeast was placed on the opposite side. After 10 min, an attraction index was calculated as (N_yeast_ – N_no yeast_)/N_total_, where N_yeast_ represents the number of *D. melanogaster* larvae in the yeast‐containing zone, N_no yeast_ represents the number of *D. melanogaster* larvae in the yeast‐free zone, and N_total_ represents the total number of larvae used.

To examine whether *L. boulardi* parasitoids are attracted to yeast volatiles, we conducted two‐choice assays in transparent acrylic cuboid boxes (length: 16 cm; width: 12 cm; height: 6 cm; Figure 1E). A plate containing yeast was placed on one side of the box, while a plate without yeast or a plate containing host larvae was placed on the opposite side. Fifteen 3‐ to 5‐day‐old female wasps were released in the center of the box. The number of parasitoids on each plate was recorded at 20 min, as the majority of individuals had made their choice by that time. The attraction index was calculated as (N_yeast_ – N_no yeast_)/N_total_ or (N_yeast_ – N_host_)/N_total_, where N_yeast_ represents the number of parasitoids on the yeast‐containing plate, N_no yeast_ represents the number of parasitoids on the yeast‐free plate, N_host_ represents the number of parasitoids on the host‐containing plate, and N_total_ represents the total number of parasitoids.

### 4.5 Wind Tunnel Experiment

The wind tunnel experimental setup was adapted from an established protocol [57] with modifications. Fifteen wasp adults (3‐ to 5‐day‐old) were placed inside a release chamber for 30 min to acclimatize prior to each test. The chamber was then positioned inside the wind tunnel, 30 cm downwind from the air intake and odor source, and parasitoid wasps were released by opening the chamber door. Each trial lasted 60 min under controlled conditions of 25°C ± 1°C, 0.2 m s^−1^ wind speed, and 60%–70% relative humidity. The positions of individual parasitoids were recorded, and their behaviors were categorized based on their location from initial take‐off to final landing. Parasitoids located in Region I (within 10 cm of the release cage) exhibited take‐off behavior; those in Region II (the central 10 cm segment of the tunnel) displayed flight behavior; individuals in Region III (within 10 cm of the odor source) demonstrated attraction behavior; and those in Region IV (on the odor source plate) exhibited landing behavior. The odor source (yeast or host larvae) was delivered on a plate placed at the upwind entrance of the tunnel at a quantity of 200 µg. An interval of at least 2 h was maintained between tests involving different odor treatments, during which the tunnel was thoroughly cleaned and dried.

### 4.6 Y‐Tube Olfactometer Bioassay

To screen the behavioral responses of female *L. boulardi* to individual yeast‐derived compounds, we used a Y‐tube olfactometer to evaluate the behavioral responses of *L. boulardi* females to yeast‐derived volatiles. The apparatus consisted of a common glass arm (7 cm in length and 1 cm in diameter) and two lateral glass arms (7 cm in length and 1 cm in diameter). Filter paper strips—one loaded with 10 µL of the different odor solutions in hexane and the other with hexane only—were placed at the distal end of each lateral arm. Purified and humidified air was introduced into each odor arm at a flow rate of 0.1 m/s, and volatile compounds were carried into the olfactometer. The system was allowed to equilibrate under ventilation for 10 min before parasitoids were released at the entrance of the common arm.

Fifteen 3‐day‐old female parasitoids were introduced into the Y‐tube and given a choice between the yeast volatile treatment and the hexane control. Each assay lasted 5 min, after which the number of parasitoids in each arm was recorded. Only parasitoids present in the two lateral arms were considered to have made a choice. The preference index was calculated as (N_volatile_ – N_hexane_)/N_total_, where N_volatile_ represents the number of parasitoids in the yeast volatile arm, N_hexane_ represents the number of parasitoids in the control arm, and N_total_ represents the total number of tested parasitoids. To control for potential directional bias, the positions of the treatment and control arms were reversed after each test, and the Y‐tube was replaced after every five assays.

### 4.7 Yeast‐Derived Volatile Chemical Analysis

The chemical composition of the yeast volatiles was analyzed using headspace gas chromatography‒mass spectrometry (HS‒GC‒MS). A mixture of 1 g of yeast and 2 mL of water was prepared, and 1 g of this mixture was transferred to a 20 mL headspace sample vial, which was then sealed with a cap for 20 min to prevent gas escape. Three independent headspace samples were prepared and analyzed.

The sealed vials were loaded into the GC‒MS headspace autosampler. Volatile extracts were separated and detected using a 7890B gas chromatograph coupled with a 5977B mass spectrometer (Agilent Technology, USA) and an HP‐5MS column (30 m × 0.25 mm × 0.25 µm; Agilent Technologies, USA). Helium carrier gas (99.999% purity) was used at a constant flow rate of 0.8 mL min^−1^ and a linear velocity of 32.4 cm s^−1^. A 1 µL aliquot of each sample was autoinjected in splitless mode at an injector temperature of 40°C. The GC program was set as follows: 40°C for 5 min, increase at 20°C min^−1^ to 200°C, and hold for 18 min. The mass spectrometer scanned a range of 20–350 m z^−1^. The MS quadrupole temperature was maintained at 250°C, and the ion source temperature was maintained at 150°C. A gain factor of 1.00 was applied, and analysis was performed in scan mode (35–550 amu) using electron ionization at 70 eV. Compounds were identified by comparison of spectral data with the NIST 17 library and validated using GC retention indices.

### 4.8 Chemicals

The following chemical compounds were used in this study:

3‐Methyl‐1‐butanol (purity >99%; CAS 123‐51‐3) was purchased from Macklin (China). All other synthetic odorants were purchased from Aladdin (China), including hexane (>98%; CAS 110‐54‐3), ethanol (>99.8%; CAS 64‐17‐5), 1‐propanol (>99%; CAS 71‐23‐8), ethyl acetate (>99%; CAS 141‐78‐6), isobutyl alcohol (>99%; CAS 78‐83‐1), 2‐methyl‐1‐butanol (>99%; CAS 137‐32‐6), ethyl isobutyrate (>99%; CAS 97‐62‐1), ethyl butyrate (>99%; CAS 105‐54‐4), ethyl isovalerate (>99%; CAS 108‐64‐5), and isoamyl acetate (>99%; CAS 123‐92‐2).

### 4.9 EAG Recording

To measure antennal sensitivity of *L. boulardi* females to yeast‐derived ethyl esters, we conducted electroantennogram (EAG) recordings on 3‐ to 5‐day‐old *L. boulardi* female adults. The head with the antennae was carefully excised and immediately attached to an antenna holder with electrode gel (Parker Laboratories, USA). The tips of the antennae were dipped into the electrode gel and gently pushed against each other so that they could stick together when they came out of the electrode gel. The prepared head was mounted by the neck onto a reference electrode consisting of an oxidized silver wire and a borosilicate pulled capillary filled with 0.9% saline solution. All test stimuli were dissolved in hexane at concentrations of 1, 10^−1^, 10^−2^, and 10^−3^ m, with hexane used as a control. Stimulations were administered in order from the lowest concentration to the highest concentration.

For each test, 10 µL of the test solution or solvent was applied to a filter paper strip (0.5 cm × 3 cm), which was then placed inside a glass Pasteur pipette. The pipette tip was inserted into a hole in the mixing tube through which a continuous stream of clean, humidified air was directed onto the antennae. Testing started when a stable baseline was established. The base of the pipette was connected to a rubber tube, and its tip was inserted into the hole on the main airflow metal tube, in which a continuous flow of 0.4 L min^−1^ was blown to the antenna. A stimulus controller was used to generate a pulsed airflow (1.8 L min^−1^) through the wide end of the Pasteur pipette, delivering volatiles to the antenna for stimulation with a 0.5 s pulse duration. Each stimulus was tested once per antenna preparation, with an interval of at least 40 s between stimuli to allow for antennal recovery. EAG signals were amplified using an impedance preamplifier (Combi probe, SYNTECH, Germany) and acquired with an Intelligent Data Acquisition Controller (IDAC‐4‐USB, SYNTECH, Germany). The signals were recorded, monitored, and analyzed using EagPro software (Syntech, Germany). EAG response amplitudes were calculated by subtracting the solvent control response.

### 4.10 Transcriptome Sequencing

Antennae from 3‐ to 5‐day‐old male and female adults were dissected in Ringer's saline solution on an ice‐cooled plate under a stereomicroscope (Leica, S9E, Germany). Total RNA was independently extracted from each sample using the RNeasy MiniKit (Qiagen, #74104, USA). cDNA library construction and paired‐end RNAseq (Illumina, NovaSeq 6000, USA) were carried out by Berry Genomics Co. Ltd. (China).

### 4.11 Genome‐Wide Identification of Odorant Receptor (OR) Genes

To identify *L. boulardi OR* genes, all published Hymenoptera OR protein sequences were downloaded from the NCBI Protein database (Last accessed: March 2, 2024) and scanned using InterProScan [82]. Sequences containing the InterPro domain “IPR004117” were selected as seed *ORs*. Transcriptomic evidence was obtained from RNA‐seq data, which were aligned to the genome assembly using STAR v2.7.10a with default parameters [83]. Braker2 v3.0.2 was then employed to integrate the aligned RNA‐seq data and the protein database for *OR* prediction within the soft‐masked genome [84]. After the duplicated genes were removed, the transmembrane‐domain (TM) structures of the resulting sequences were predicted by CCTOP (https://cctop.ttk.hu, HUNGARY) [85]. Owing to rapid evolution or the presence of multiple introns, some putative olfactory receptors represented fragmented or incomplete *OR* genes. Sequences shorter than 100 amino acids and containing fewer than 3 TMs were manually removed. All *L. boulardi OR* genes identified through this process were designated “*LbouORX*”, where X represents a unique Arabic numeral (e.g., *LbouOR1*, *LbouOR2*, etc.).

### 4.12 Phylogenetic and Expression Profile Analysis

We performed a phylogenetic analysis of ORs from four hymenopteran species: *L. boulardi*, *A. mellifera*, *N. vitripennis*, and *H. saltator*. The translated amino acid sequences of complete *OR* genes were aligned using MAFFT v7.520 with auto strategy parameters [86]. Poorly aligned regions were removed using trimAl v1.4 [87]. Phylogenetic analysis was conducted using IQ‐TREE2 under the maximum likelihood (ML) method [88], with the parameters “‐m MFP ‐b 1000 ‐alrt 1000”. The phylogenetic tree was inferred with 1000 bootstrap replicates and visualized using iTOL v6 (https://itol.embl.de) [89]. To characterize the differentially expressed *OR* genes, we analyzed RNA sequencing data from female and male antennae. Expression levels were quantified as normalized transcripts per million (TPM) for each gene using Salmon v1.10.3 [90].

### 4.13 RNA Interference (RNAi)

To investigate the functional roles of *LbouOR167* and *LbouOR136* in detecting yeast‐derived ethyl esters, we performed RNAi‐mediated knockdown of these genes. Approximately 400 bp fragments of the target genes were amplified by PCR from the cDNA. Double‐stranded RNA (dsRNA) was synthesized using the T7 RiboMAX Express RNAi System Kit (Promega, USA) according to the manufacturer's instructions. *GFP* was used to generate control dsRNA (*dsGFP*). The primers used are listed in Table S6. The reaction mixture was incubated at 37°C for 4 h, heated at 70°C for 10 min, and then allowed to anneal at room temperature for 20 min. The dsRNA was subsequently treated with RNase and DNase I enzymes (Vazyme, China) to remove template residues and purified using isopropanol. Purified dsRNA was quantified with a NanoDrop 2000 spectrophotometer (Thermo Fisher Scientific, USA). Approximately 20 nL of dsRNA (concentration: 5 µg µL^−1^) was injected into *L. boulardi* prepupae using an Eppendorf FemtoJet 4i device (Eppendorf, Germany) with the following parameters: injection pressure = 900 hPa and injection time = 0.15 s.

### 4.14 Quantitative Real‐Time PCR (qRT‒PCR)

Total RNA was extracted from *L. boulardi* tissues (e.g., antennae) using a RNeasy Mini Kit (Qiagen, USA) and reverse transcribed into cDNA using HiScript III RT SuperMix for qPCR (Vazyme, China) according to the manufacturer's protocol. qRT‒PCR was performed on a QuantStudio3 Real‐Time PCR System (Thermo Fisher Scientific, USA) with a ChamQ SYBR qPCR Master Mix Kit (Vazyme, China). The expression levels of target genes were normalized to those of *tubulin* mRNA, and relative expression was determined using the 2 ^−ΔΔCt^ method. All the primers used for qRT‒PCR are listed in Table S6.

### 4.15 Fluorescent Calcium Assay

To functionally validate the responses of *LbouOR167* and *LbouOR136* to yeast‐derived ethyl esters, we performed calcium imaging in Human Embryonic Kidney 293 (HEK293) cells. The coding regions of *LbouOR167* and *LbouOR136* were amplified from *L. boulardi* antennal cDNA using the primers listed in Table S6. The resulting fragments were subsequently cloned and inserted into the pcDNA3.1 (+) vector using NotI and EcoRI enzymes (NEB, USA). Site‐directed mutagenesis was performed using the Mut Express II Fast Mutagenesis Kit V2 (Vazyme, China), with the primers listed in Table S6. Plasmids carrying *LbouOR* genes were transformed into *Escherichia coli* for amplification and purification using the EndoFree Plasmid Midi Kit (CWBIO, China).

HEK293 cells were cultured in Dulbecco's modified Eagle medium (Thermo Fisher Scientific, USA) supplemented with 10% fetal bovine serum (Thermo Fisher Scientific, USA) at 37°C under 5% CO_2_. Cultured HEK293 cells were cotransfected with 1 µg of *LbouOR* plasmid and 3 µg of *LbouOrco* plasmid using Lipofectamine 3000 (Invitrogen, USA) in 6‐well cell culture plates (Thermo Fisher Scientific, USA). Transfected cells were transferred to an 8‐well chambered coverglass (Thermo Fisher Scientific, USA) and incubated with loading buffer (Hanks’ balanced salt solution containing 1 µm Fluo‐4 AM and 0.2% pluronic F127) for 30 min. The loading buffer was then removed, and the cells were rinsed twice with Hanks’ balanced salt solution. Odorant‐induced receptor activation was measured after the addition of odorants using a Zeiss LSM 800 confocal microscope. Test odorants were dissolved in DMSO at five concentrations (10^−7^, 10^−6^, 10^−5^, 10^−4^, and 10^−3^ m) for sigmoidal dose‒response curve fitting. The relative change in fluorescence (ΔF/F_0_) was used to quantify the responses, where F_0_ represents the baseline mean cell fluorescence and ΔF represents the difference between the peak mean cell fluorescence induced by the test odorants.

### 4.16 In Situ Hybridization

To determine whether *LbouOR167* and *LbouOR136* are co‐expressed within the same olfactory sensilla, we performed two‐color fluorescent in situ hybridization on female *L. boulardi* antennae following a previously reported protocol with modifications [91]. Briefly, gene‐specific probes were synthesized from the open‐reading frames of *LbouORs* using the primers listed in Table S6. Digoxigenin (Dig)‐labeled LbouOR136 probes and biotin (Bio)‐labeled LbouOR167 probes were synthesized using a DIG RNA labeling Kit (SP6/T7) (Roche, Mannheim, Germany) with Dig‐NTP or Bio‐NTP labeling mixtures (Roche, Mannheim, Germany) according to the manufacturer's instructions. Freshly dissected antennae from 3‐ to 5‐day‐old *L. boulardi* females were embedded in JUNG tissue freezing medium (Leica, Nussloch, Germany). Sections approximately 12 µm thick were prepared using a microtome (Leica CM1950, Germany) at −22°C and mounted on Superfrost Plus microscope slides (Thermo Fisher Scientific, USA). The sections were fixed in 4% paraformaldehyde for 30 min, treated with 0.2 m HCl for 10 min, and incubated in 50% deionized formamide/5× SSC for 10 min. Each slide was covered with 100 µL of hybridization solution containing labeled antisense RNA probes, sealed with a coverslip, and incubated overnight at 60°C in a humidified chamber with 50% formamide. The slides were subsequently washed in 0.1×SSC at 60°C and treated with 1% blocking solution (Roche, Germany). The blocked slides were incubated for 60 min with anti‐digoxigenin (Roche, Germany) to detect the Dig‐labeled probes and with streptavidin‐HRP (ABclonal, China) to detect the labeled probes. Hybridization signals were visualized by incubating sections for 30 min with Vector Red Reagents (red) using the VECTOR Red Alkaline Phosphatase (Red AP) Substrate Kit (Vector Laboratories, USA), followed by incubation for 10 min with Biotinyl Tyramide Working Solution (green) using a tyramide signal amplification (TSA) kit (ABclonal, China) at room temperature to detect Dig‐ and Bio‐labeled probes, respectively. Fluorescence images were acquired using a Zeiss LSM 800 confocal microscope.

### 4.17 Structural Prediction and Molecular Docking

Predicted monomer structures of LbouOR167 and LbouOR136 were generated using AlphaFold2 (DeepMind, UK) [54]. Structural comparisons between LbouOR167 and LbouOR136 were performed using PyMOL 3.0 (Schrödinger, USA; downloaded from https://pymol.org). Model quality was validated with ERRAT and PROCHECK (https://saves.mbi.ucla.edu/). The 3D structures of the four target compounds—ethyl acetate (PubChem CID: 8857), ethyl isobutyrate (CID: 7342), ethyl butyrate (CID: 7762), and ethyl isovalerate (CID: 7945)—were retrieved from the PubChem database (https://pubchem.ncbi.nlm.nih.gov/). Structural files of proteins and compounds were converted to the Protein Data Bank format using Open Babel v3.1.1 for subsequent molecular docking [92].

Molecular docking of the two ORs with the four target compounds was performed using AutoDock v4.2.6 [93]. The top ligand poses were selected based on the lowest binding affinity. Receptor–ligand interactions were analyzed and visualized using the Protein–Ligand Interaction Profiler (PLIP; https://plip‐tool.biotec.tu‐dresden.de/plip‐web/plip/index) to identify putative binding pockets in the modeled structures [94]. Structural visualization and interaction characterization were conducted using PyMOL 3.0. Putative binding pockets in the OR models were further identified using CASTpFold (https://cfold.bme.uic.edu/castpfold/) [95].

### 4.18 Single Sensillum Recording

Seven‐ to 10‐day‐old *D. melanogaster* male flies were immobilized in truncated 200‐µL plastic pipette tips (Axygen, USA), with their antennae exposed and secured using plasticine under a stereomicroscope. A reference glass electrode (World Precision Instruments, USA) was inserted into the compound eye, while a tungsten microelectrode (tip sharpened to 0.5 µm) was inserted into the base of the T1 sensilla. Odorants were dissolved in hexane, after which 10 µL of diluted odorant solutions (10^−4^, 10^−3^, 10^−2^, 10^−1^, and 1 m) were adsorbed onto filter strips (1 × 3 cm) placed inside Pasteur pipettes (150 mm; Witeg, Germany), with hexane used as a solvent control. Odor delivery was controlled using a CS‐55 stimulator (Syntech, Germany), which generated 300‐ms odor pulses superimposed on a continuous 30 mL s^−1^ air stream passing through an activated carbon filter. The signals were amplified with an IDAC‐4 amplifier (Syntech, Germany), digitized, and recorded using Autospike 3.9 (Syntech, Germany). Each stimulus was recorded as a random odor with an interstimulus interval of at least 1 min to prevent sensory adaptation. The change in spikes per second (spikes s^−1^) was calculated as follows: spikes s^−1^ = (number of spikes within 1 s after stimulation) – (number of spikes within 1 s before stimulation).

### 4.19 Statistical analysis

Statistical analyses were performed using GraphPad Prism v8.0 (GraphPad Software, USA) and SPSS 26 (IBM, USA). The normality of the data distribution was assessed with the Shapiro–Wilk test, and the homogeneity of variance was evaluated using Bartlett's chi‐square test. For the oviposition index, preference index, and attraction index, comparisons against a theoretical value of 0 (no preference) were conducted using Wilcoxon's signed‐rank test. For comparisons between two groups under parametric assumptions, two‐tailed unpaired Student's *t* tests were applied. The Mann–Whitney *U*‐test was used for nonparametric comparisons of two groups. ANOVA followed by Tukey's multiple comparisons test was used to compare means across multiple groups under parametric conditions. Detailed descriptions of the statistical methods, significance thresholds, and specific tests used are provided in the figure legends. The data are presented as the means ± standard errors of the mean (SEMs). Statistically significant differences (*p* < 0.05) among multiple groups are indicated by different letters. For pairwise comparisons, significance levels are indicated as follows: ^*^ *p* < 0.05; ^**^ *p* < 0.01; ^***^ *p* < 0.001.

## Author Contributions

J.H. conceived the project. J.H. and L.P. designed and directed the study. Y.L., T.F., S.Z., and L.P. performed behavioral experiments. Y.L., W.S., H.G., and J.C. conducted electrophysiology experiments. Y.L., L.Y., and L.P. performed qRT‐PCR and RNAi experiments. Y.L., W.S., and Z.D. performed OR annotation, phylogenetic, and expression analysis. Y.L. and W.S. performed modeling and docking analyses. Y.L. and L.P. performed in situ hybridization and fluorescent calcium assays. J.H., L.P., Y.L., and W.S. interpreted the data. J.H., L.P., Y.L., and W.S. wrote the manuscript. All authors have read and approved the manuscript submission.

## Conflicts of Interest

The authors declare no conflicts of interest.

## Supporting information




**Supporting File 1**: advs75253‐sup‐0001‐SuppMat.docx.

**Supporting File 2**: advs75253‐sup‐0002‐Tables S1–S6.xlsx.

## Data Availability

The data that support the findings of this study are openly available in NCBI database at https://www.ncbi.nlm.nih.gov/, reference number PRJNA1100901.
