# Supplementary material for: Microbial Odorant Detection Guides Drosophila Parasitoids Seeking Hosts in Fermenting Fruits
Source: Adv Sci (Weinh). 2026 Apr 13;13(39):e75253. doi: 10.1002/advs.75253 (PMC13334941; doi:10.1002/advs.75253)
Supplement: Supplementary file 1 — Supporting File 1: advs75253‐sup‐0001‐SuppMat.docx. [file ADVS-13-e75253-s002.docx]

Supporting Information

**Microbial Odorant Detection Guides *Drosophila* Parasitoids Seeking Hosts in Fermenting Fruits**

*Yueqi Lu, Lan Pang*, Wenqi Shi, Ting Feng, Zhi Dong, Yifeng Sheng, Sicong Zhou, Longtao Yu, Hao Guo, Ying Wang, Jiani Chen, Jianhua Huang**


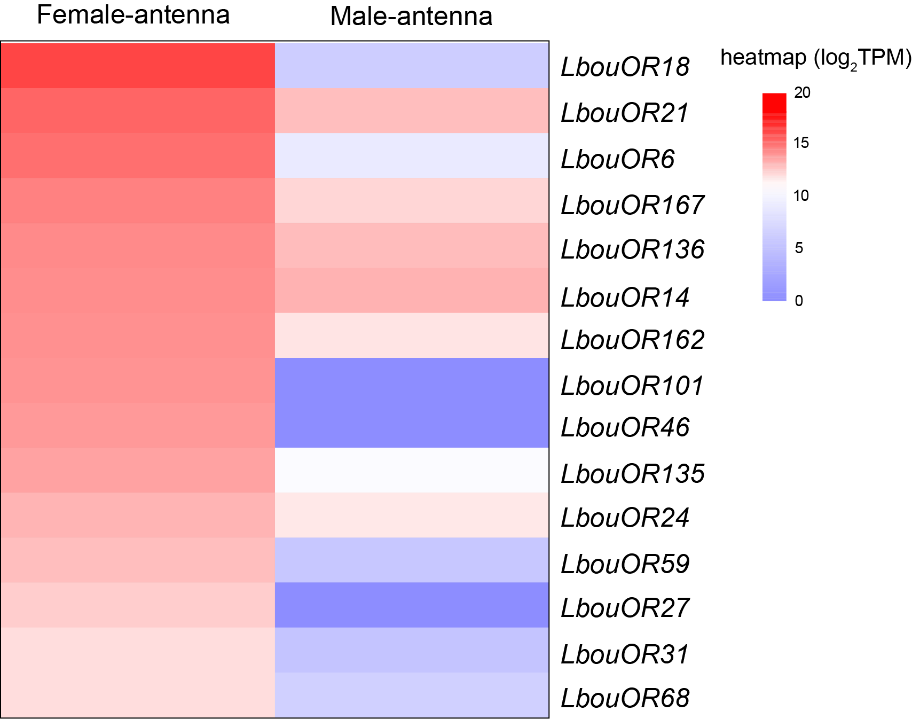


**Figure S1. Female antennae-biased *LbouOR* genes in the 9-exon subfamily.**

The genes are arranged according to their expression levels in female antennae.


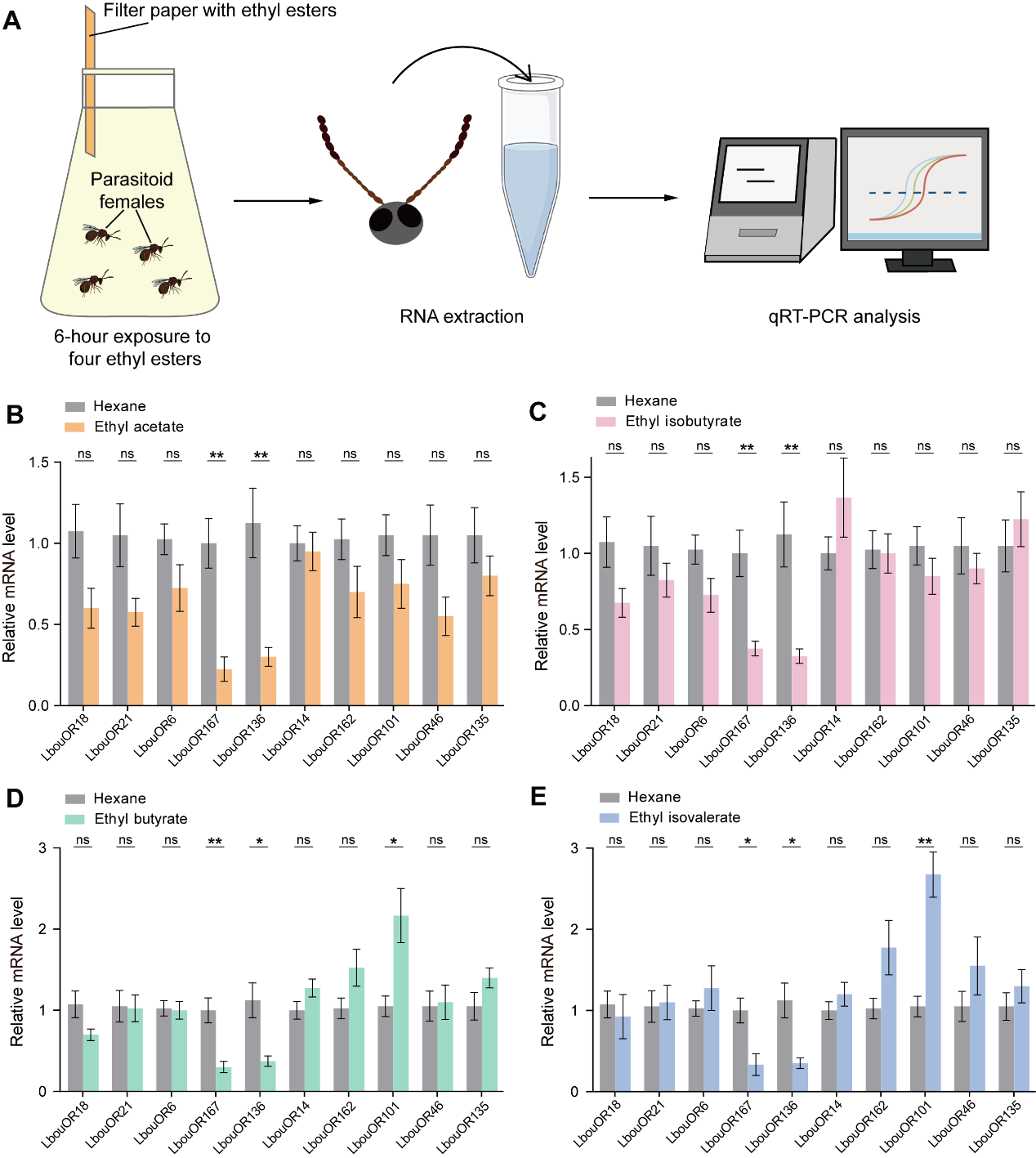


**Figure S2. Changes in the expression of *LbouORs* following exposure to yeast-derived ethyl esters.**

**(A)** Schematic diagram of the experimental procedure: RNA was extracted from the heads and antennae of female *L. boulardi* parasitoid wasps after 6 h of exposure to four ethyl ester compounds; subsequent qRT‒PCR was performed to detect changes in the expression of the *LbouOR* genes. **(B-E)** Relative expression levels of *LbouORs* after 6 h of exposure to 1 M solutions of **(B)** ethyl acetate, **(C)** ethyl isobutyrate, **(D)** ethyl butyrate, and **(E)** ethyl isovalerate. Female wasps exposed to hexane served as the control. Four biological replicates were performed per odorant treatment. Data represent the means ± SEMs. Significance was determined by an unpaired two-tailed Student’s *t* test (**p* < 0.05; ***p* < 0.01; ns, not significant).


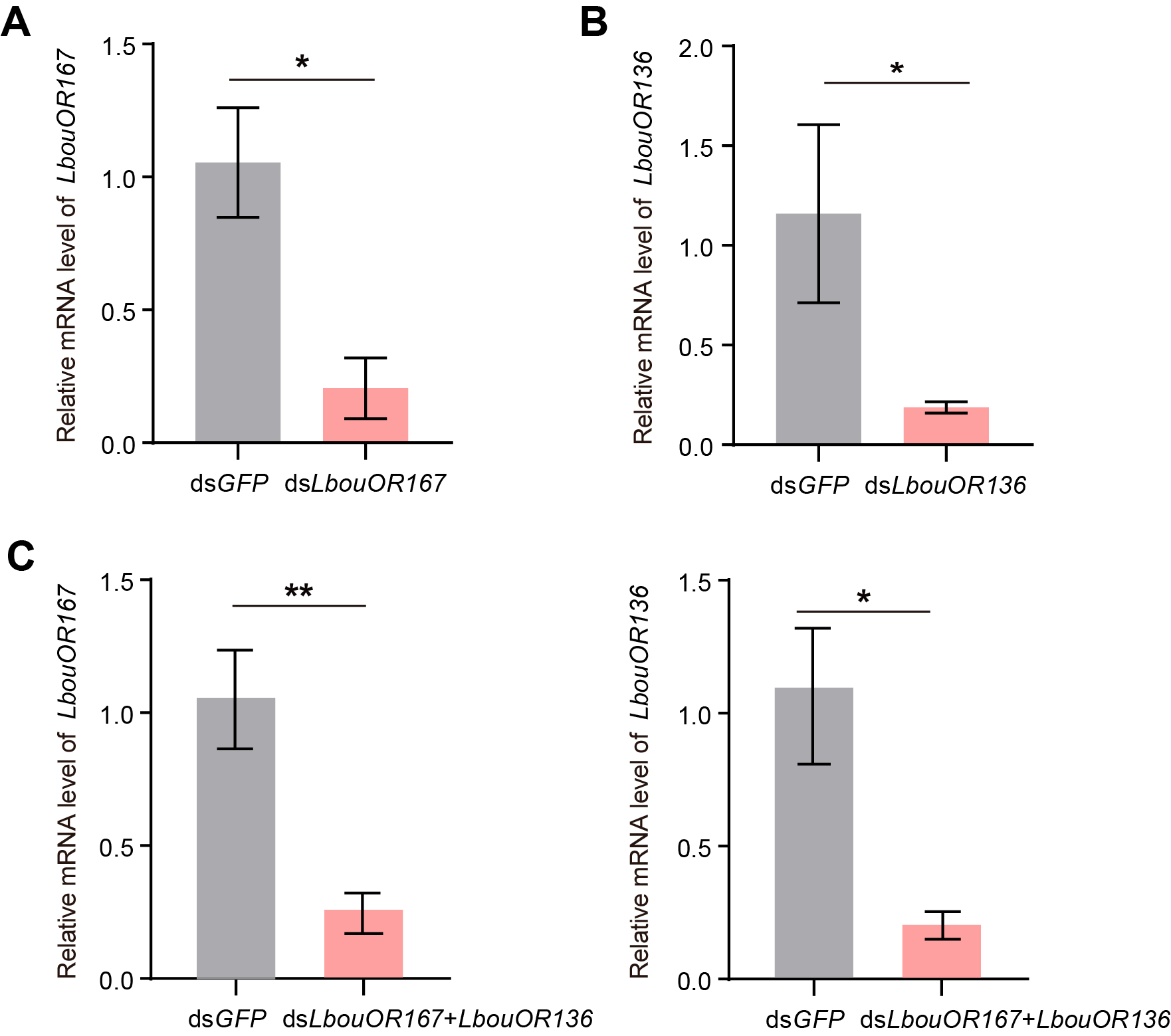


**Figure S3. RNAi efficiency of *LbouOR167* and *LbouOR136*.**

**(A)** Relative expression level of *LbouOR167* in *L. boulardi* females after injection of ds*LbouOR167*. **(B)** Relative expression level of *LbouOR136* in *L. boulardi* females after injection of ds*LbouOR136*. **(C)** Relative expression levels of *LbouOR167* and *LbouOR136* in *L. boulardi* females after coinjection of ds*LbouOR167* and ds*LbouOR136*. At least 3 biological replicates were performed per treatment. Data represent the means ± SEMs. Significance was determined by two-sided unpaired Student’s *t* test (**p* < 0.05; ***p* < 0.01).


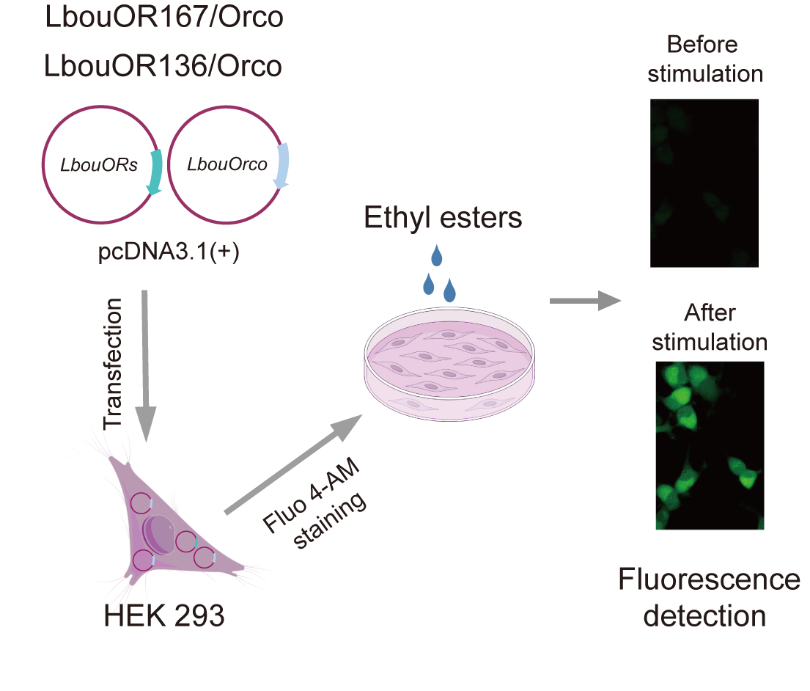


**Figure S4. Schematic diagram of the HEK293 cell-based calcium imaging assay.**

Diagram illustrating the experimental setup for fluorescent calcium imaging in HEK293 cells expressing *L. boulardi* odorant receptors. Cells were cotransfected with *LbouOR* and *LbouOrco* plasmids, loaded with a Fluo-4 AM calcium indicator, and exposed to ethyl ester compounds, and real-time calcium flux was monitored using confocal microscopy.


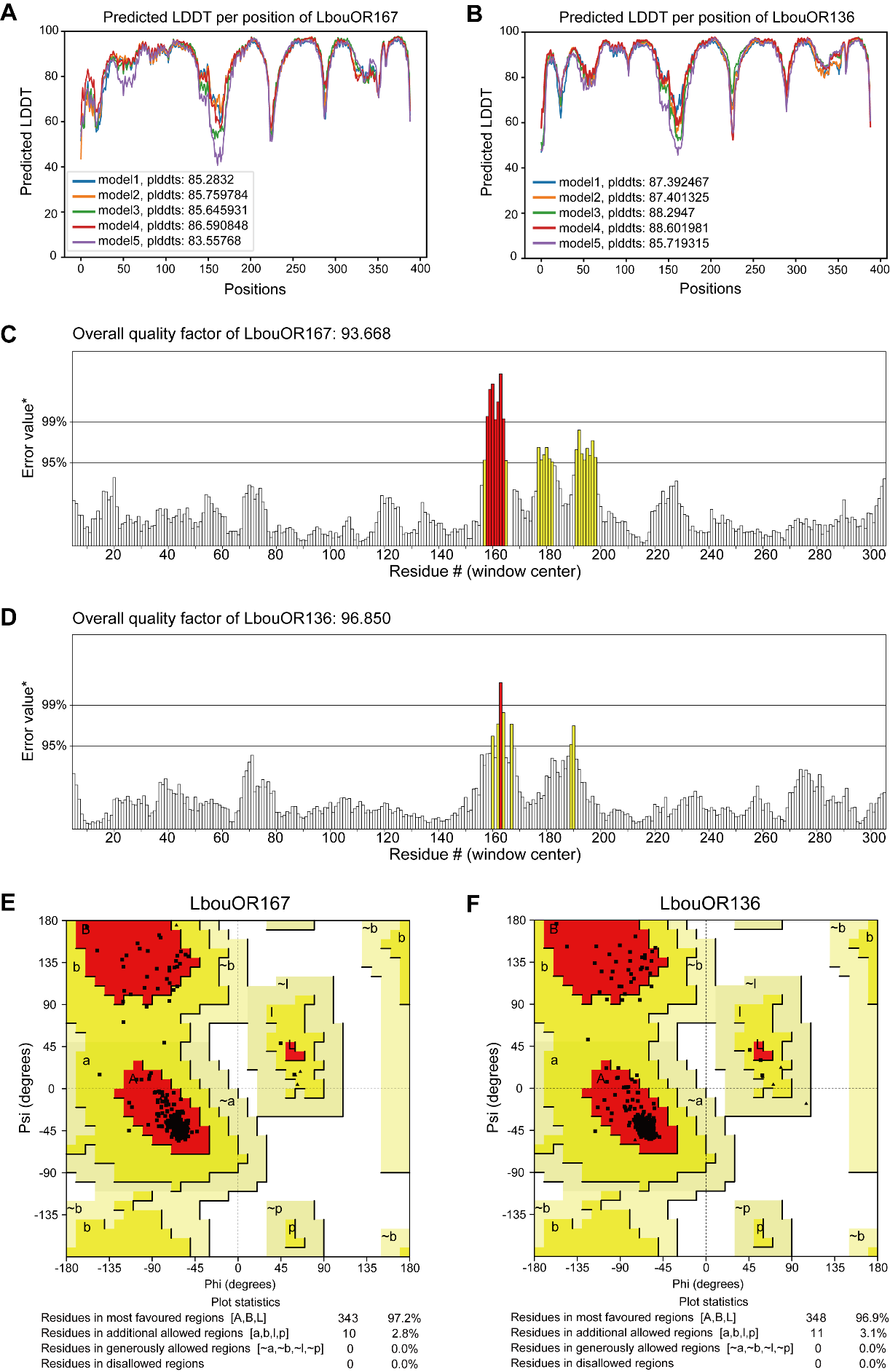


**Figure S5. Validation of the predicted structural models for LbouOR167 and LbouOR136.**

**(A and B)** Five predicted 3D models of LbouOR167 and LbouOR136 with corresponding pLDDT scores, which reflect confidence in local structural accuracy. Higher pLDDT values indicate greater residue-level reliability. The median pLDDT score for each model is indicated in the lower left corner. (**C and D)** ERRAT analysis of the LbouOR167 and LbouOR136 models. The overall quality factor reflects nonbonded atomic interactions, with higher scores indicating improved stereochemical reliability. **(E and F)** Ramachandran plots and residue distribution statistics from PROCHECK analysis. Regions are defined as follows: most favored (bright red; A, B, L), additionally allowed (bright yellow; a, b, l, p), generously allowed (light yellow; ~a, ~b, ~l, ~p), and disallowed (white). The black dots represent individual amino acid residues. A reliable model should have >90% nonglycine and nonproline residues in the most favored regions.


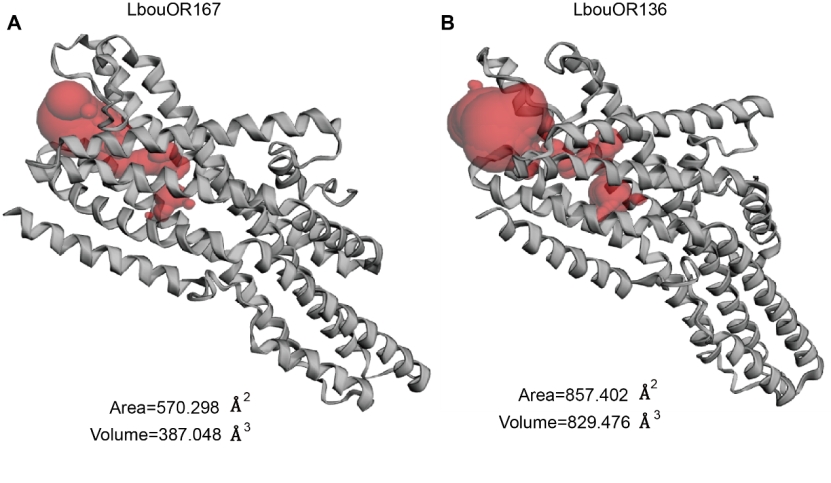


**Figure S6. Predicted ligand-binding pockets of LbouOR167 and LbouOR136.**

Overview of the predicted binding pocket (highlighted in red) in the **(A)**, LbouOR167 and **(B)**, LbouOR136 structural models identified by CASTpFold.


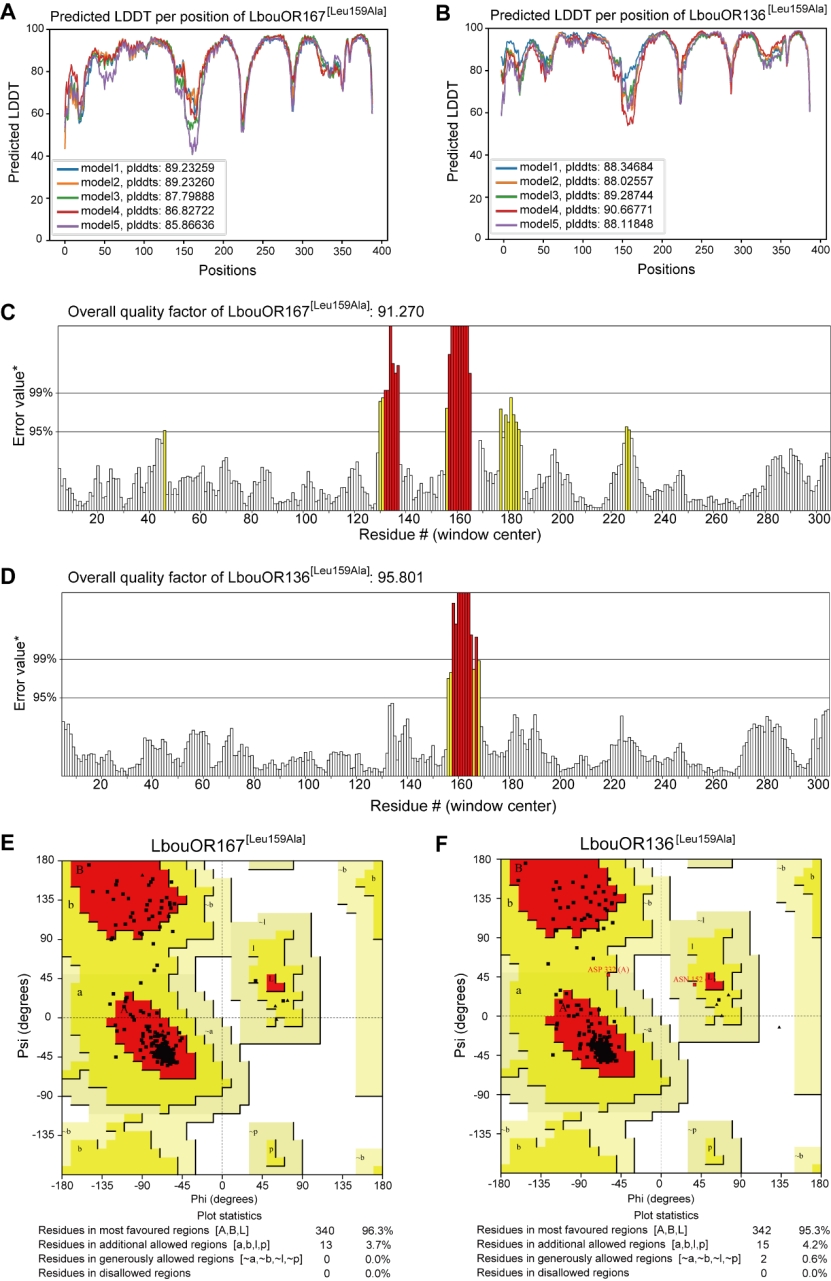


**Figure S7. Structural validation of the LbouOR167^[Leu159Ala]^ and LbouOR136^[Leu159Ala]^ mutant models.**

**(A and B)** Five predicted 3D models of **(A)** LbouOR167^[Leu159Ala]^ and **(B)** LbouOR136^[Leu159Ala]^ corresponding to pLDDT scores. **(C and D)** ERRAT analysis of the **(C)** LbouOR167^[Leu159Ala]^ and **(D)** LbouOR136^[Leu159Ala]^ models. **(E and F)** Ramachandran plots and residue distribution statistics from PROCHECK analysis of **(E)** LbouOR167^[Leu159Ala]^ and **(F)** LbouOR136^[Leu159Ala]^.


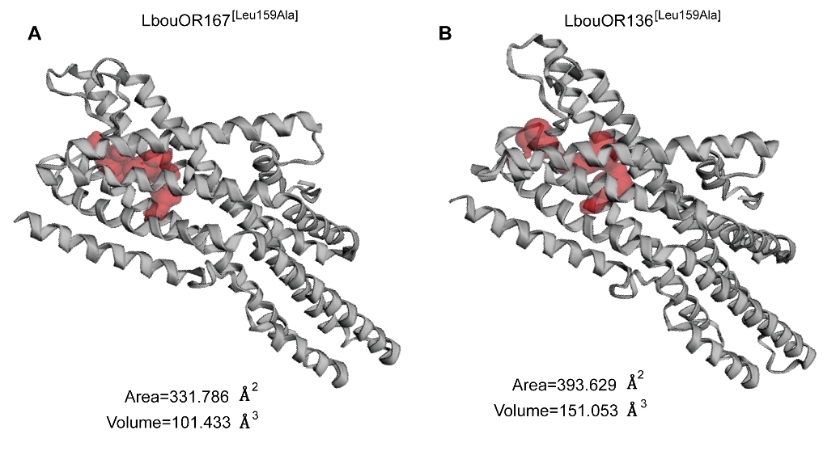


**Figure S8. Predicted ligand-binding pockets of LbouOR167^[Leu159Ala]^ and LbouOR136^[Leu159Ala]^.**

Overview of the predicted binding pocket (highlighted in red) in the **(A)** LbouOR167^[Leu159Ala]^ and **(B)** LbouOR136^[Leu159Ala]^ structural models, identified by CASTpFold.


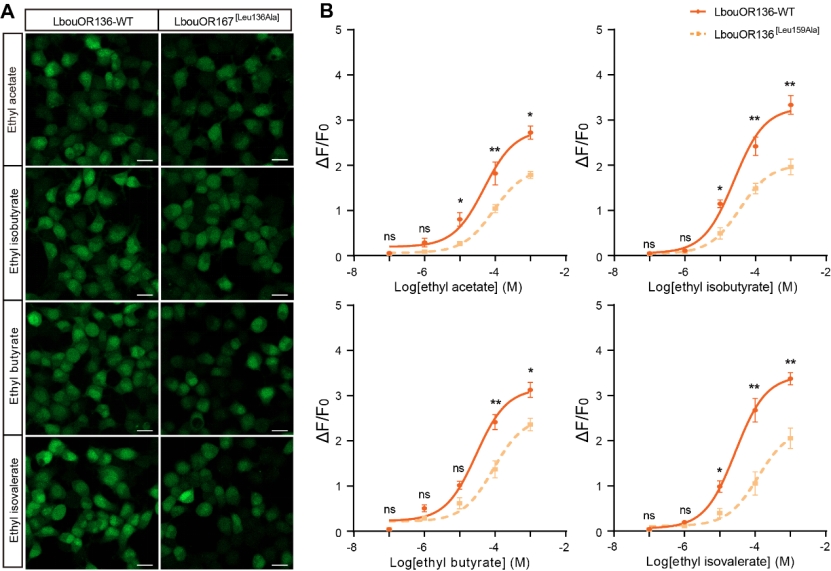


**Figure S9. Calcium imaging of HEK-293 cells expressing wild-type and mutant *LbouOR136*.**

**(A)** Representative fluorescence images of HEK293 cells expressing *LbouOR136* or *LbouOR136^[Leu159Ala]^* after stimulation with 10^-3^ M ethyl ester compounds. Scale bar: 20 μm. **(B)** Dose‒response curves of LbouOR136 and LbouOR136^[Leu159Ala]^ to ethyl ester compounds. At least 4 biological replicates were performed. Data represent the means ± SEMs. Significance was determined by an unpaired two-tailed Student’s *t* test (**p* < 0.05; ***p* < 0.01; ns, not significant).


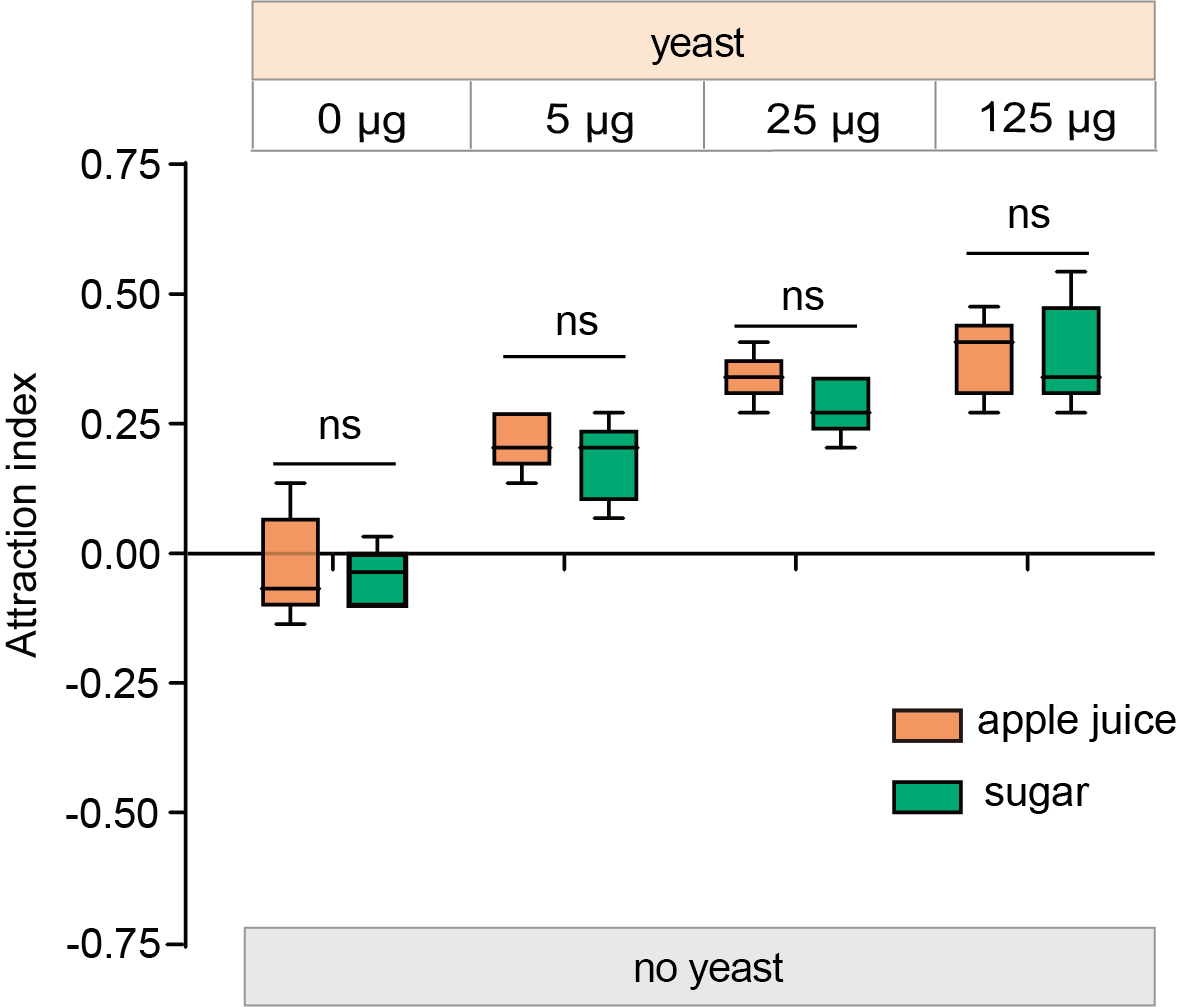


**Figure S10. Attraction indices of female *L. boulardi* reared on different diets to varying yeast quantities.**

Wasps were maintained on either apple juice agar or sugar-only diets. No significant difference in attraction was observed between the two diet groups across all yeast concentrations tested. The box plots represent the median (centerline), interquartile (box), and full data range (whiskers), respectively. Five biological replicates were performed. Statistical significance was assessed using two-way ANOVA followed by Tukey's multiple comparisons test (ns, not significant).

**Supplementary Table legends**

**Table S1.** Identified odorant receptor genes in *L. boulardi*.

**Table S2.** Numbers of hymenopteran OR genes in different subfamilies.

**Table S3.** Differentially expressed odorant receptor genes in male and female antennae of *L. boulardi*.

**Table S4.** Predicted binding pocket residues in LbouOR167 and LbouOR136 identified by CASTp analysis.

**Table S5.** Key hydrophobic residues mediating interactions between LbouORs and ethyl esters.

**Table S6.** Primer sequences used in this study.
